# Diagnostically analyzing ^1^H NMR spectra of sub-types in chaetoglobosins for dereplication†

**DOI:** 10.1039/c9ra10434h

**Published:** 2020-01-09

**Authors:** Chen Lin, Jian-chun Qin, Yong-gang Zhang, Gang Ding

**Affiliations:** Key Laboratory of Bioactive Substances and Resources Utilization of Chinese Herbal Medicine, Ministry of Education, Institute of Medicinal Plant Development, Chinese Academy of Medical Sciences, Peking Union Medical College Beijing 100193 P. R. China gding@implad.ac.cn; Zhengzhou Key Laboratory of Synthetic Biology of Natural Products, Henan Joint International Research Laboratory of Drug Discovery of Small Molecules, Huanghe Science and Technology College Zhengzhou Henan 450063 P. R. China; College of Plant Sciences, Jilin University Changchun Jilin 130062 P. R. China; Biology Institute, Qilu University of Technology (Shandong Academy of Sciences) Jinan 250103 Shandong Province P. R. China zhangygcq@163.com

## Abstract

^1^H-NMR spectra provide abundant diagnostic information including chemical shift values, splitting patterns, coupling constants, and integrals. Thus some key functional groups, and even planar structures could be elucidated on the basis of carefully analyzing the corresponding ^1^H NMR spectrum. In this paper, the different sub-types of chaetoglobosins are classified according to the structural features, of which the ^1^H NMR spectra are systematically summed up. Thus diagnostically analyzing the ^1^H-NMR spectra could identify possible sub-types of chaetoglobosins, which could be used for dereplication. According to the analysis of this report, it implies that different new sub-types or new sub-type combinations in the key skeleton of chaetoglobosins might exist in nature. More importantly, dereplication based on ^1^H NMR spectral analysis will not only provide a useful approach to determine the chaetoglobosins structures quickly, but also could set a good example for structural dereplication of other NPs.

## Abstract 2

^1^H-NMR spectra provide abundant diagnostic information including chemical shift values, splitting patterns, coupling constants, and integrals.

## Graphical abstract

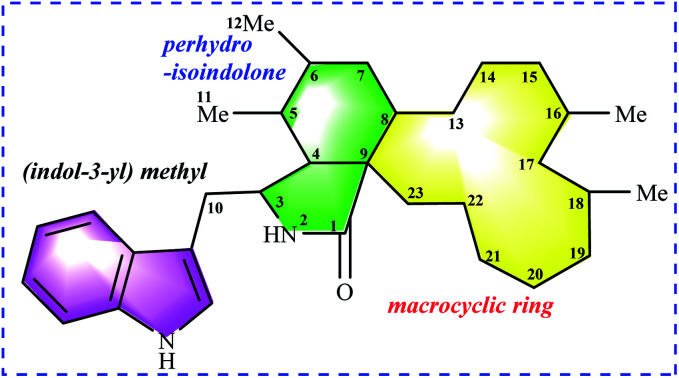

## 1. Introduction

Natural products (NPs) play a pivotal role in drug discovery and development, and many drugs in prescriptions directly or indirectly originate from NPs.^1,2^ Nowadays purification of NPs is not a laborious task due to the combination of different chromatographic technologies, whereas structural elucidation of NPs is still a manual job, which needs much time (even several months) to characterize the complex structures. To date, several techniques including HR-MS (High Resolution Mass), MS^n^, NMR (Nuclear Magnetic Resonance) spectroscopy, X-ray diffraction, and even bioinformatics approaches are used to elucidate the structures of NPs. HR-MS can give exact molecular weight leading to the molecular formula, and provides unsaturation degrees of target NPs; MS^n^ can produce precursor and product ions, which help to deduce possible structural fragments. Combination of HR-MS and MS^n^ techniques is akin to piecing together a complex jigsaw puzzle by assembling the molecular weight, molecular formula and structural fragments to elucidate structures. Most of the time this approach could not establish the planar structure especially for complex structures; X-ray diffraction can obtain the direct structural information including planar, relative and absolute configurations, whereas not all NPs could be grown to be a suitable crystal for X-ray diffraction experiment. Recently, bioinformatics approaches were also used to predict the possible substrates of secondary metabolites such as polyketide synthases (PKSs) or nonribosomal peptide synthetases (NRPSs) biosynthesized compounds.^3^ Considering the different post-modification reactions in the biosynthetic pathways, using bioinformatic way to elucidate the NPs structures is still not a reliable method now. NMR techniques such as 1D NMR (^1^H, and ^13^C NMR spectra), and 2D NMR such as HMQC, ^1^H ^1^H-COSY, HMBC, and NOESY spectra are the main tools to elucidate the structures of different NPs. Actually, ^1^H NMR spectral analysis is the first and key step in the structural elucidation of NPs. Compared with other NMR spectra, ^1^H NMR spectrum has a wealth of diagnostic information including chemical shift values, splitting patterns, coupling constants, and integrals, which reflect the structural features.^4^

Chemical shift values are the direct reflection of different functionalities in the structure. For example, usually the chemical shift values of aromatic or olefinic proton are 6.0–9.5 or 4.5–7.5 ppm, respectively.^5^ If there are electron withdrawing groups (such as keto-group) near the aromatic proton or olefinic proton, the chemical shift values of these protons nearby will be shifted to be more down-fielded, *vice versa*. Thus according to different chemical shift values, the possible functional groups in the structure could be predicted.

Splitting patterns can tell us how many protons connecting the corresponding groups. If a proton is a triplet (t) or a doublet doublet (dd), implying that a –CH_2_– unit or two different protons might be connected near by the proton.

Analysis of coupling constants can help determine the configuration of corresponding protons with others. Usually big coupling constant indicates *trans*-configuration, *vice versa*. For example, if the coupling constant of olefinic protons is over 15.0 Hz, indicating the *Z*-configuration of the double bond, otherwise *E*-configuration.

Integrals could give the proton numbers of a natural product, but the intergrals are different in different deuterated solvents. Usually, the integrals (in DMSO-*d*_6_) could reflect the total proton numbers including exchangeable protons.

Then detailed analysis of chemical shift values, splitting patterns, coupling constants, and integrals in the ^1^H NMR spectrum can characterize structural fragments, and even establish the possible planar structures. If the deduced structures are known compounds by searching different data bases such as SCIfinder, Dictionary of Natural Products (DNP) or others, there is no need to process the next steps (^13^C, HMQC, ^1^H ^1^H-COSY, HMBC, and NOESY spectra) further, which can save a lot of time and resources. In this paper, the different sub-types of chaetoglobosins are classified according to the structural features, of which the ^1^H NMR spectral are systematically summed up. Finally, two examples are given in details for dereplication by diagnostically analyzing the ^1^H NMR spectra on this member of mycotoxins (other eight examples are included in ESI†).

## 2. Results and discussion

### 2.1 Structural features of chaetoglobosins

**Fig. 1:**
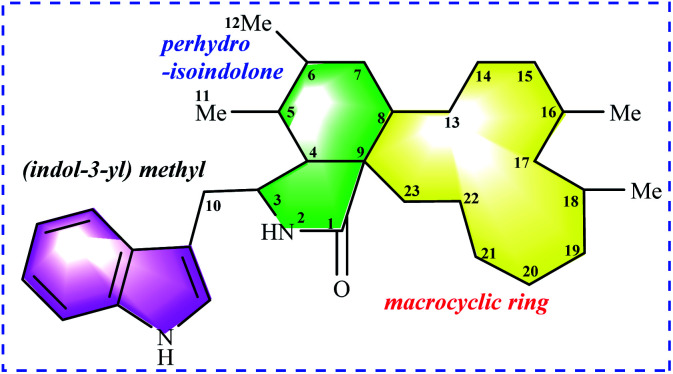
Chaetoglobosin nucleus: (indol-3-yl) methyl unit, perhydro-isoindolone moiety, and macrocyclic ring.

Chaetoglobosins are a large member of mycotoxins biosynthesized by a PKS−NRPS hybrid megasynthetase.^6,7^ The structural features of these compounds are a highly substituted perhydro-isoindolone moiety bearing an (indol-3-yl) methyl group to which typically a macrocyclic ring – is fused (Fig. 1). The different substitutions, connectivity patterns and oxygenation sites on the perhydro-isoindolone moiety and macrocycle ring increase the chemical diversity. Up to date, 56 analogs (See ESI†) containing the key nucleus of chaetoglobosin (perhydro-isoindolone moiety and macrocycle ring Fig. 1) were isolated different fungi mainly from *Chaetomium* spp.^7,8^

**Fig. 2:**
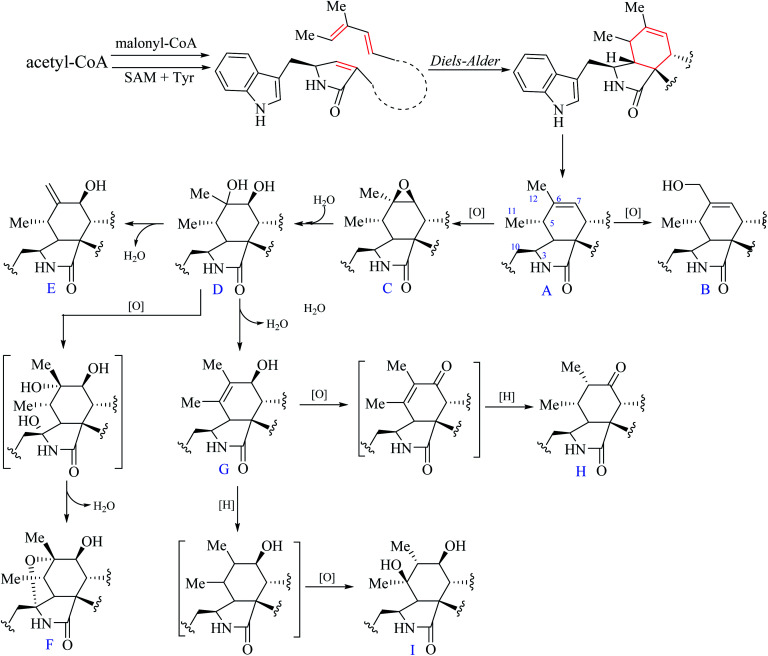
Nine sub-types in the perhydro-isoindolone moiety.

**Fig. 3:**
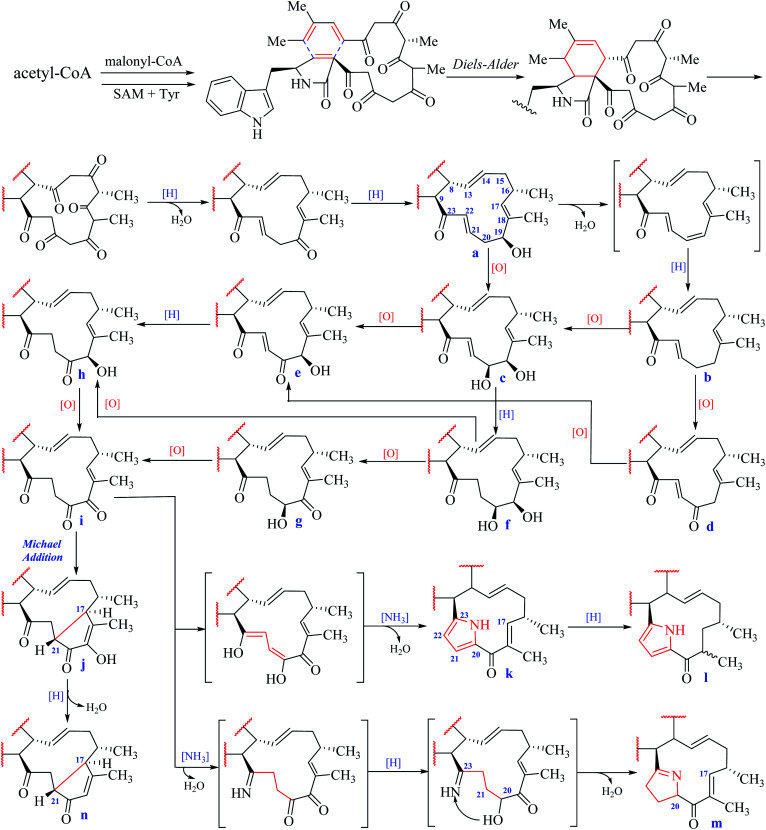
Fourteen sub-types in the macrocyclic ring.

According to the structural features of chaetoglobosins, 9 sub-types in the perhydro-isoindolone moiety are summed up on account of the different variations at C-3, C-5, C-6, C-7 and C-12 (Fig. 2), whereas 14 sub-types in the macrocycle ring are classified according to the diverse variations at C-17, C-18, C-19, C-20, C-21, C-22 and C-23 (Fig. 3).

### 2.2 ^1^H NMR spectral characteristics of perhydro-isoindolone moiety

From the postulated biogenetic pathway of chaetoglobosins, the Diels–Alder reaction forms the key cyclohexene ring (C-4-C-5-C-6-C-7-C-8-C-9), and then a series of post-modification reactions mainly oxygenations at C-3, C-5, C-6, C-7 and C-12 construct nine sub-types in the perhydro-isoindolone part. This leads to chemical environment variations of different protons in the perhydro-isoindolone ring, which produces different chemical shift values, splitting patterns, coupling constants (of H-7, –CH_2_-10, CH_3_-11, and CH_3_-12), and different integrals. Thus according to different chemical shift values, splitting patterns, coupling constants, and integrals of those protons, it could, in return, deduce the structural fragments on the perhydro-isoindolone moiety.

#### A-type

This sub-type contains seven analogs namely chaetoglobosins J,^9^ T,^10^ penochalasin G,^11^ cytoglobsin D,^12^ prochaetoglobosins I-II^13^ and armochaetoglobin K.^14^ 11-Me and 12-Me in these compounds are observed as a doublet (chemical shift value under 1.50 ppm) and a singlet connected on a double bond (chemical shift value over 1.50 ppm), respectively. The olefinic proton (H-7) usually as a broad singlet peak (the dihedral angle between H-7/H-8 close to 90°) is observed in the low-field (5.00–6.00 ppm), and the signal of H-8 is located around 2.00–3.00 ppm with a big coupling constant (*J*_8, 13_ ≈ 10 Hz). Usually, –CH_2_-10 is displayed as two doublet doublets (2.50–3.00 ppm), and the chemical shift value of H-3 (multiplet) is often around 3.00–4.00 ppm, whereas the protons H-4 and H-5 is located between 2.00–3.00 ppm as a doublet doublet, and a multiplet, respectively.

#### B-type

This sub-type of chaetoglobosins includes 3 analogs cytoglobsin E,^12^ armochaetoglobosin L,^14^ and armochaetoglobin W,^15^ which is the corresponding oxidized product of the 12-Me to –CH_2_O-12 from sub-type A. The chemical shift value of this group (–CH_2_O–12 as a broad singlet peak) is at 3.80–4.20 ppm. Thus B-type analogs have an additional oxymethylene unit and a less singlet methyl compared with that of A-type ones.

#### C-type

Fourteen compounds (chaetogolobosins A,^16^ C,^17^ F,^17^ and U,^18^ prochaetoglobosins III-IV,^13^ 20-dihydrochaetogolobosins A,^19^ penochalasins A,^20^ D-F,^11^ armochaetoglobins P-Q^14^ and armochaetoglobin Y^15^) are included in this sub-type with the double bond (C-6/C-7) oxygenated to be the corresponding epoxide group compared with A-type. In the ^1^H NMR spectrum, the 11-Me and 12-Me groups as a doublet and a singlet are located at the upfield zone (1.00–1.50 ppm), respectively. The chemical shift values of 12-Me in these A and C-types are different. Usually 12-Me in A-type is over 1.50 ppm, and 12-Me in C-type is under 1.50 ppm due to its connection with a double bond. In addition, owing to the effect of the epoxide group, the proton signal H-7 (*J*_7, 8_ = 6.0 Hz) as a doublet is located between 2.50–3.00 ppm.

#### D-type

This sub-type of compound is the hydrolytic product of C-type, in which the epoxide group is hydrolyzed to be the corresponding diol moiety. Six compounds chaetoglobosins Q-R,^10^ armochaetoglobin S,^15^ 7-*O*-acetylarmochaetoglobin S,^15^ armochaetoglobin U,^15^ and armochaetoglobin X^15^ are contained in this sub-type. The main difference between C and D-types is that the chemical shift value of H-7 in D-type is located in a more down-field (3.10–3.60 ppm) with a big coupling constant (*J*_7, 8_ = 10.0–12.0 Hz) compared with that of C-type.

#### E-type

One new terminal double bond at C-6/C-12 in E-type is formed by loss of a molecule of H_2_O from D-type. This sub-type of chaetoglobosins has two additional olefinic protons as broad singlet peaks with chemical shift values around 5.00 ppm in the ^1^H NMR spectrum. Thus the singlet methyl group (12-Me) is absent in the ^1^H NMR spectrum compared with that of D-type. There are nine analogs (cytoglobsoins A, B, F, G,^12^ chaetoglobosin D,^17^ isochaetoglobosin D,^17^ chaetoglobosin F_ex_,^19^ penochalasin C,^20^ and armochaetoglobin R^14^) in the E-type.

#### F-type

This sub-type of chaetoglobosins is the oxidized and dehydrated products of D-type, which possesses a unique ether bond between C-3 and C-6 forming an additional furan ring in the perhydro-isoindolone moiety. This variation leads to both H-10a and H-10b to be two doublets (at 3.00–3.50 ppm), which is distinct from all other analogs. In addition, the peak of H-4 is changed to be a singlet, and the NMR signal of H-3 is absent compared with D-type. F-type possesses two analogs armochaetoglobin M^14^ and chaetoglobosin W.^21^

#### G-type

Thirteen compounds are contained in this sub-type of chaetogolobosins including chaetoglobosins B,^16^ E,^17^ G,^9^ O,^22^ V_1_,^21^ V_b_,^23^ V_2_,^24^ prochaetogolobosin III_ed_,^24^ cytoglobosin C,^12^ armochaetoglobins N–O,^14^ penochalasin B,^20^ and armochaetoglobin Z.^15^ G-type analogs are originated from D-type by loss of a molecule of H_2_O, which shapes a double bond at C-5 and C-6. Thus both 11-Me and 12-Me display as singlet peaks with chemical shift values at 1.00–2.00 ppm different from all other sub-types, and the oxymethine H-7 as a doublet peak (*J*_7, 8_ = 10.0 Hz) is located at 3.50–4.50 ppm in the ^1^H NMR spectrum, which usually leads to the peak of H-8 to be a triplet (t) or a doublet doublet (dd) with big coupling constants (*J*_7, 8; 8,13_ = 7–10.0 Hz).

#### H-type

This sub-type might come from G-type by oxidizing C-7 to be a keto-group, and reducing the double bond C-5 and C-6 to be two methine units. The diagnostic characteristics of this type in ^1^H NMR is that both 11-Me and 12-Me are doublet peaks in high-field zone (1.00–2.00 ppm) different from all other sub-type chaetoglobosins, and the C-7 is further oxygenated to be a keto carbonyl group leading to the chemical shift value of H-8 to be down-fielded (3.65 ppm, doublet, *J* = 9.4 Hz). Only one compound chaetoglobosin Y is included in H-type.^25^

#### I-type

The last sub-type (armochaetoglobin V) is the isomer of D-type with hydroxylization at C-5 not C-6.^15^ These changes lead to different splitting patterns, and coupling constants of H-4 and H-7 in these two sub-types. H-4 is a multiplet in D-type not a broad doublet in I-type, whereas H-7 is a doublet in D-type not a doublet doublet (dd) in I-type (in CD_3_OD).

### 2.3 ^1^H NMR characteristics of macrocyclic ring

In the macrocyclic ring of chaetoglobosins, C-8, C-9, C-13, C-14, C-16, 16-Me, and 18-Me are usually not changed. Considering different oxygenation degrees at C-19 and C-20, the double bond formation at C-21/C-22, the carbon–carbon connectivity at C-17/C-21, or pyrrole ring formed by C-20, 21, 22 and 23 in the macrocylic ring, 14 sub-types (a–n) are summed up (Fig. 3), which are distinct from each other according to their diagnostic ^1^H NMR features.

#### a-type

This sub type of compounds contain only an analog namely chaetoglobosin T,^10^ which might be originated from its precursor by reducing the keto group C-19 to the corresponding hydroxyl group with chemical shift value at 4.38 ppm (–OCH–19, multiplet in CDCl_3_) (Fig. 3). A doublet methyl and a singlet methyl in the ^1^H NMR spectrum belong to 16-Me (0.95, d, *J* = 7.0 Hz) and 18-Me (1.52, br. d, *J* = 1.0 Hz), respectively. The olefinic protons H-13 and H-14 are located at 5.96 (dd) and 5.10 (ddd). The methylene group –CH_2_-15 and methine –CH-16 are located in the high-field (2.0–2.50 ppm) as multiplet peaks. In addition, this sub-type possesses a specific a, β-unsaturated *trans*-double bond at C-21=C-22–C=O-23 with chemical shift values at 6.80 ppm (m, H-21) and 6.89 ppm (d, *J* = 16.0 Hz, H-22).

#### b-type

Three analogs prochaetoglobosins I,^13^ IV^13^ and chaetoglobosin V_2_ (ref. 24) are included in this sub-type, which might be the dehydrated and reductive products from a-type (Fig. 3). Thus this type of chaetobglobosins is absent an oxymethine unit (H-19) with one more methylene unit in the high-fielded zone compared with that of a-type analogs.

#### c-type

This type contains 4 compounds namely cytoglobosins B-D^12^ and 20-dihydrochaetoglobosin A^19^ with an additional hydroxyl group anchored at C-20 compared with a-type. Thus an extra oxymethine proton signal (–CHO–20) with chemical shift values at 4.00–4.50 ppm is observed, which lead to the signal of olefinic proton H-21 to be as a doublet doublet (dd) not a multiplet (m) or a doublet triplet (dt) present in a and b-types. This change makes c-type have a less methylene unit than that of a-type analogs.

#### d-type

There are 3 analogs including prochaetoglobosins II, III^13^ and III_ed_^24^ in this type with an additional keto carbonyl group at C-20 compared with b-type. This variation brings the chemical shift value of H-22 to the low-field (>7.50 ppm) as a doublet, which distinguishes it from a, b, c-types. In addition, the signal of methylene-19 is observed between 3.50–4.00 ppm.

#### e-type

This sub-type of chaetoglobosins might be originated from c-type by oxidizing C-20 or from d-type by oxidizing C-19. Thus compared with sub-type d, e-type has a more hydroxyl unit at C-19 with chemical shift values at 5.00–6.00 ppm as a singlet or doublet (in DMSO-*d*_6_), whereas compared with c-type, e-type has remarkably different chemical shift values and splitting patterns at H-19, H-21 and H-22. Six analogs chaetogolobosins A, B, D,^16^ J,^9^ Q and R^10^ are contained in this type.

#### f-type

This type of compounds is the reductive products of c-type at the double bond C-21/22. Thus the chemical shift values of H-19 and H-20 are relatively in upfield zone compared with those of c-type (c-type: H-19 at 4.00 ppm, br.s, H-20 at 4.50 ppm, br.s; f-type: H-19 at 3.39 ppm, br.s, H-20 at 3.20 ppm, br.s). The corresponding double bond protons H-21 and H-22 are disappeared in this type, and two additional methylene units are observed in upfield zone. Only one compound cytoglobosin G^11^ is found in f-type.

#### g-type

Nine analogs are contained in this sub-type including chaetogolobosins E-F,^17^ F_ex_,^19^ Y,^25^ W,^21^ armochaetoglobin S,^15^ 7-*O*-acetylarmochaetoglobin S,^15^ armochaetoglobin V^15^ and armochaetoglobin W.^15^ The 19-OH was further oxidized to the corresponding keto carbonyl group compared with f-type, and the chemical shift value of H-20 is down-fielded to be 4.50–500 ppm as triplet (in CDCl_3_). Owing to the effect of α, β-unsaturated keto carbonyl group (C-17–C-18–C=O-19), the chemical shift value of H-17 in the ^1^H NMR spectrum is in down-fielded zone between 6.00–6.50 ppm compared with the a–f types (<5.70 ppm).

#### h-type

This sub-type chaetoglobosins could be biosynthesized from e-type through reducing the double bond C-21/22 or from f-type by oxygenating C-20. So compared with the ^1^H NMR spectrum of e-type, one less double bond is observed in h-type with two additional methylenes in high-field, whereas compared with that of f-type, h-type has two less protons, and the oxymethine unit (H-19) is transferred to be 4.00–4.50 ppm as a singlet (s) or a doublet (in DMSO-*d*_6_). Five compounds chaetoglobosin O,^22^ cytochalasin F,^12^ penochalasins E–G^11^ are contained in this sub-type.

#### i-type

From the biosynthetic view, this type of compounds is the further oxygenated products of g or h-type analogs with two keto carbonyl group found at C-19 and C-20. Thus i-type chaetoglobosins possesses a less oxymethine than that of g or h-types. Five compounds cytochalasin E,^12^ chaetogolobosins C, G, isochaetoglobosin D^17^ and armochaetoglobin U^15^ are included in this sub-type.

#### j-type

This type of analogs is the intramolecular Michael-addition products of i-type chaetoglobosins at C-17 and C-21. Thus the olefinic proton of H-17 is disappeared, and the chemical shift value of 18-Me is down-fielded to 1.90–2.00 ppm in the ^1^H NMR spectrum, which is distinct from all other analogs. In addition, there is a unique enol-hydroxyl signal with chemical shift value at 9.05 ppm (s, in DMSO-*d*_6_). This type contains five compounds named chaetogolobosins U,^18^ V_1_,^21^ Vb,^23^ cytochalasin A,^12^ and armochaetoglobin X.^15^

#### k-type

This type of chaetoglobosins possesses a unusual pyrrole ring formed by C-20, 21, 22 and 23, which might be originated from i-type analogs by coupling with one molecule of NH_3_ and then losing two molecules of H_2_O. Thus in the ^1^H NMR spectrum of k-type, there are two coupled olefinic protons with chemical shift values around 6.30–7.50 ppm (H-21 and H-22), and an additional free proton is located at 10.0–12.0 ppm (–NH–, br.s, in CDCl_3_). In addition, due to the effect of 19-keto carbonyl group, the chemical shift value of H-17 and 18-Me is down-fielded around 5.50–6.00 ppm and 1.90 ppm, respectively. Six analogs including penochalasins A–C,^20^ and armochaetoglobins K–M^14^ are contained in this sub-type.

#### l-type

Five analogs armochaetoglobins N–R^14^ are in this of sub-type, which is the reductive product of k-type at the olefinic carbons C-17 and C-18. Thus this type of compounds has an additional methyl group as doublet peak in the high field around 1.00–1.50 ppm and a less double bond proton (H-17) compared with k-type.

#### m-type

This sub-type compounds might be originated from i-type through reacting with NH_3_ to shape a 3,4-dihydro-2*H*-pyrrole ring. Compared with i-type analogs, j-type has an additional methine signal (H-20) with chemical shift value around 4.19 ppm as doublet doublet triplet (ddt) peak. In addition, the chemical shift value of olefinic proton H-17 in these two types is also different. There is only one analog penochalasin D^11^ contained in this sub-type.

#### n-type

Two analogs armochaetoglobins Y^15^ and Z^15^ are included in this sub-type, which might be biogenetically come from j-type by reduction and dehydration reactions. The main difference between these two sub-types is that n-type has an additional singlet (H-19) with chemical shift value at 5.90–6.10 ppm (s, in CD_3_OD), whereas j-type analogs have a free hydroxyl group at 9.05 ppm (s, in DMSO-*d*_6_).

According to the classification of different sub-types in chaetoglobosins, theoretically, there exist 126 analogs (9 × 14), which include all the 56 known chaetoglobosins possessing the nucleus: indol-3-yl methyl unit, perhydro-isoindolone moiety, and macrocyclic ring (Fig. 1). All these known chaetoglobosins are the different assembly of A–I types in perhydro-isoindolone moiety combined with a–n types in macrocyclic ring. Thus according to the ^1^H-NMR rules of sub-types, analyzing the ^1^H-NMR spectra of chaetoglobosins can deduce the corresponding structures fast. The purpose of the following 10 examples (eight examples are included in ESI†) is to explain how to analyze ^1^H NMR spectral to elucidate the structures of chaetoglobosins, and then to dereplicate known analogs.

### 2.4 Analysis of ^1^H NMR spectra to dereplicate known chaetoglobosins

**Fig. 4:**
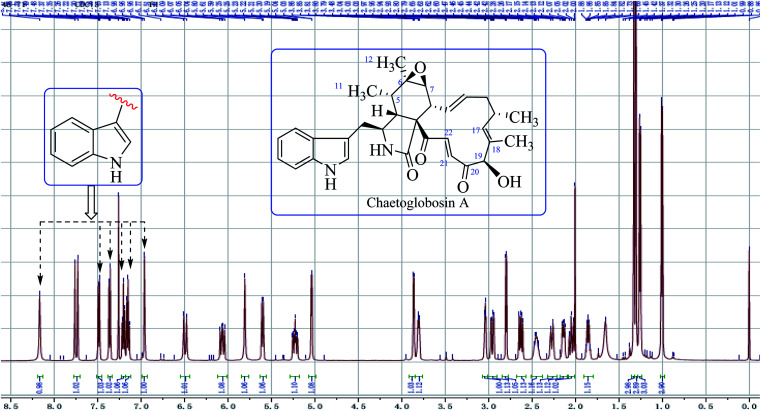
The full spectrum of chaetoglobosin A in CDCl_3_.

**Fig. 5:**
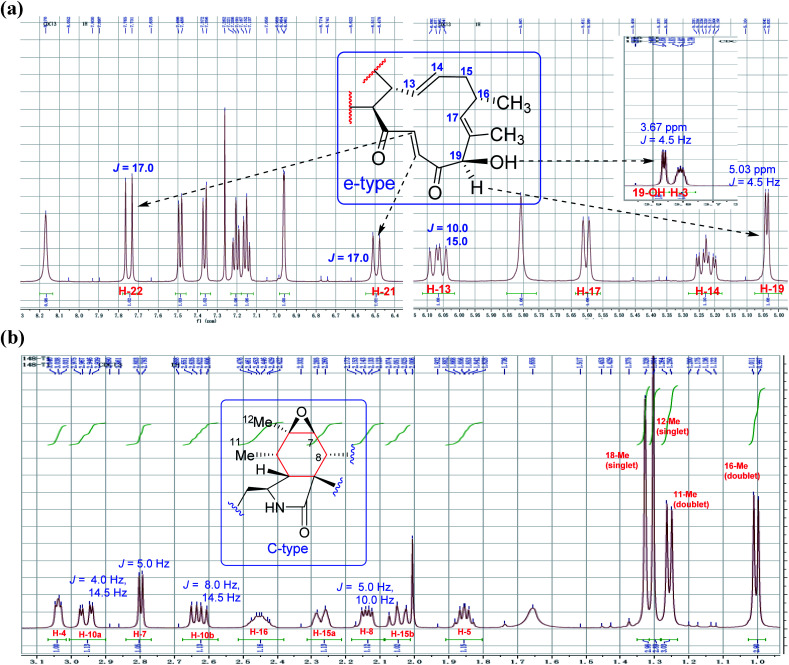
(a and b) Expanded ^1^H NMR spectrum of chaetoglobosin A (1).

**Fig. 6:**
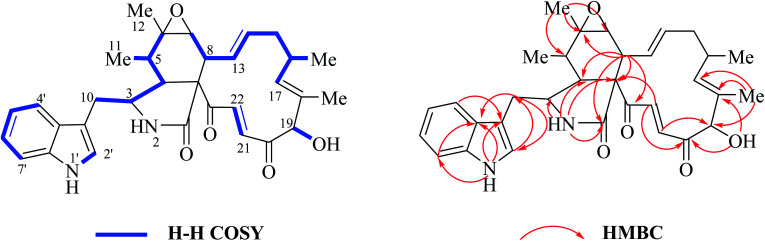
^1^H-^1^H COSY, and key HMBC and NOESY of chaetoglobosin A (1).

Chaetoglobsin A (1) = (C-type + e-type)The full ^1^H NMR spectrum (in CDCl_3_) of analog (1) is depicted in Fig. 4. From the down-fielded zone, there existed typically a set of indole ring proton signals. A pair of *trans*-olefinic protons with chemical shift values at 7.75 (d, *J* = 17.0 Hz, H-22) and 6.50 ppm (d, *J* = 17.0 Hz, H-21) were observed, which implied the existence of an α, β-unsaturated ketone group (20C=O–21CH=22CH). The signal at 5.04 ppm (*J* = 4.5 Hz, H-19) coupled the one at 3.86 ppm (*J* = 4.5 Hz, 19-OH) revealed the structural features of 19-OH and 20-keto-group in the macro-ring. In addition, the olefinic protons H-13/H-14/H-17 together with the 16-Me (doublet) and 18-Me (singlet) in high field were observed in the ^1^H NMR spectrum, which confirms that this compound must possess the e-type of macrocyclic ring (Fig. 5a and b). The proton signals of H-7 (*δ*_H_ = 2.80, d, *J* = 5.0 Hz) along with the 11-Me (*δ*_H_ = 1.26, d) and 12-Me (*δ*_H_ = 1.30, s) in the up-field indicated the existence of a peroxide at C-6 and C-7. In addition, the H-3, H-4, H-5, H-8, and –CH_2_-10 were also were observed in the ^1^H NMR spectrum, which completely matched the C-type in perhydro-isoindolone moiety. Thus detailed analysis of the ^1^H NMR spectrum revealed that this chaetoglobosin belongs to the combination of e-type plus C-type (Fig. 5a and b). Searching the structural types of all known analogs, it demonstrated that only does chaetoglobosin A possess the combination of e-type and C-types.^16^ Finally, this conclusion was confirmed by 2D NMR spectra (Fig. 6).

The ^1^H NMR spectra analysis of other known analogues (2–9) were provided in ESI.†

### 2.5 Analysis of ^1^H NMR spectra to target new chaetoglobosin analog

Analyzing the ^1^H NMR spectrum of chaetoglobosins can discriminate the differently combinatory types of the 56 known analogs. Thus new characteristics or combinations in the ^1^H NMR spectra different from those of 56 analogs will imply new chaetoglobosins. Recently, we isolated a series chaetoglobosins from *OE::CgLaeA C. globosum* (CBS148.51).^26^ Detailed analyzing the ^1^H NMR spectra of these compounds revealed that one of analogs contained different combination of sub-types, and it implied that this compound might be a new chaetoglobosin. This hypothesis was further supported by 2D NMR spectra. The following example will explain the characteristics of ^1^H NMR spectrum of this new compound with different sub-types found in this analog.Chaetoglobosin Z (10) = (D-type + e-type)

**Fig. 7:**
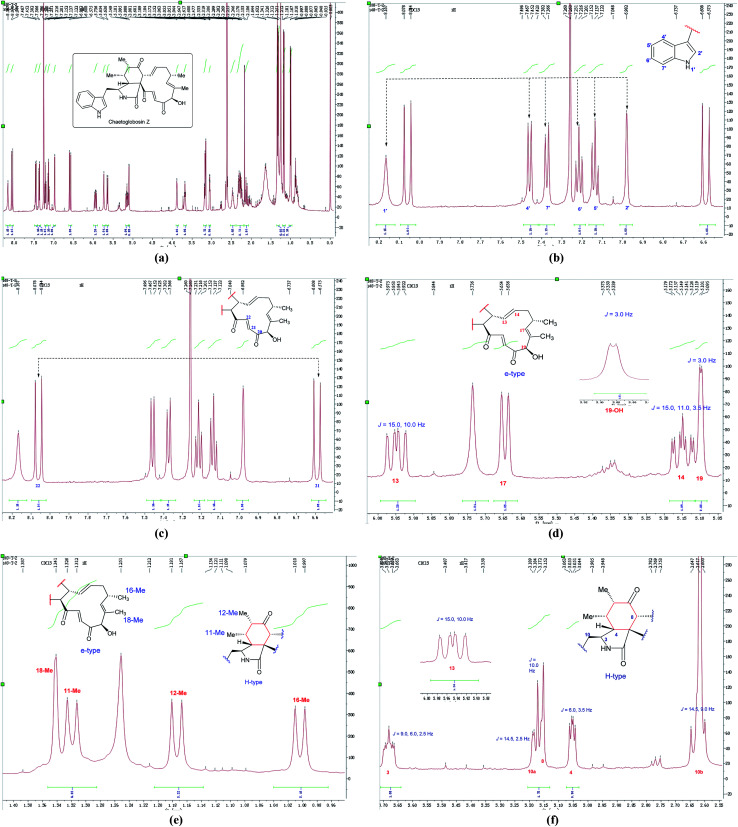
(a) The full spectrum of chaetoglobosin Z (10) in CDCl_3_. (b–f) Expanded ^1^H NMR spectrum of chaetoglobosin Z (10) in CDCl_3_.

In the ^1^H NMR spectrum of chaetoglobosin Z (10) (Fig. 7a), the diagnostic proton signals of an indole-ring are observed in the down-fielded zone (Fig. 7b). The *trans*-olefinic protons with chemical shift values at 8.06 ppm (H-22, *J* = 16.5 Hz) and 6.59 ppm (H-21, *J* = 16.5 Hz) implied that C-20 must possess a keto-group leading the chemical shift value of H-22 to be significant down-field (Fig. 7c). Three signals of olefinic protons including H-13 (5.95 ppm, dd, *J* = 15.0, 10.0 Hz), H-14 (5.15 ppm, ddd, *J* = 15.0, 11.0, 3.5 Hz) and H-17 (d) are observed between 5.00–6.00 ppm. Considering the chemical shift value of H-17 (5.65 ppm), this revealed that C-19 do not possess the keto-group (if C-19 is oxidized to be corresponding keto-group, which will lead the chemical shift value of H-17 to be more down-fielded, < 6.00 ppm). The oxymethine proton (5.10 ppm, *J* = 3.5 Hz) coupled with the corresponding proton (3.88 ppm, *J* = 3.5 Hz) confirms that the C-19 might contain a free hydroxyl group same as those of chaetoglobosins A, B, J and others (Fig. 7d). On accounting of the fact that signals of 16-Me (doublet) and 18-Me (singlet) are not changed in the upfield zone, together considering the information above-mentioned, it implied that chaetoglobosin Z (10) must contain the e-type in macrocyclic ring.

**Fig. 8:**
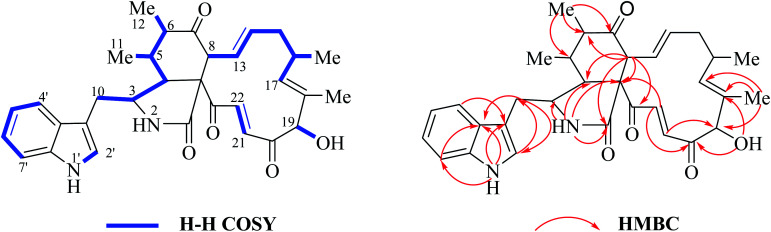
^1^H–^1^H COSY, and key HMBC and NOESY of chaetoglobosin Z (10).

Two doublet peaks (11-Me and 12-Me) except 16-Me (doublet) and 18-Me (singlet) in the high field are observed (Fig. 7e), which implies that the C-5 and C-6 must be two methine groups. In addition, the chemical shift value of H-8 is down-fielded to 3.16 ppm as a doublet peak (*J* = 10.0 Hz) coupled with olefinic proton H-13 (5.95 ppm, dd, *J* = 15.0, 10.0 Hz) (Fig. 6e), which denotes that the C-7 must be oxidized to be the corresponding keto-group. The splitting patterns of H-3, H-4 and –CH_2_-10 are not changed, and the chemical shift values of these protons (H-3, H-4 and –CH_2_-10) show small differences (Fig. 7f), which reveals that perhydro-isoindolone moiety in chaetoglobosin Z (10) must be D-type. Thus, chaetoglobosin Z (10) contains the combination of D-type plus e-type, which is different from those found in other 56 known chaetoglobosins, implying that this compound might be a new chaetoglobosin analog. Finally, the planar, and relative configuration of this new compound were determined by 2D NMR spectra (Fig. 8).^26^

## 3. Conclusion

In conclusion, compared with other techniques, ^1^H NMR spectrum contains a wealth of diagnostic information, and analysis of ^1^H NMR spectrum is the first and key step, which can infer the structural fragments and even characterize the possibly planar structures. In this report, the different sub-types of perhydro-isoindolone moiety (A–I types, 9 sub-types) and of macrocyclic ring (a–n, 14 sub-types) in the chaetoglobosins are classified according to their possibly biogenetic pathway and structural features. Attempts to summarize the relationship between structural features and ^1^H NMR spectral are tried, which can dereplicate known analogs, and target new ones. According to the structural features of chaetoglobosins in this paper, new chaetoglobosins with different sub-type combinations or new sub-types are waiting to be found in future. More importantly, the results in this report will not only provide a useful approach to determine the different chaetoglobosins structures fast, but also could set a good example for other NPs structural elucidation.

## 4. Experimental section

### 4.1 Samples

The compounds in this report have been isolated and characterized previously in the authors' laboratories.^26–28^

### 4.2 NMR Spectroscopy

^1^H, ^13^C and 2D NMR data were acquired with a Bruker 600 spectrometer using solvent signals (DMSO-*d*_6_: *δ*_H_ 2.50/*δ*_C_ 39.9; CDCl_3_: *δ*_H_ 7.26/*δ*_C_ 77.6) as references.

## Conflicts of interest

There are no conflicts of interest to declare.

## Supplementary Material

RA-010-C9RA10434H-s001
