# Supplementary material for: Diagnostically analyzing 1H NMR spectra of sub-types in chaetoglobosins for dereplication
Source: RSC Adv. 2020 Jan 9;10(4):1946–55. doi: 10.1039/c9ra10434h (PMC9047530; doi:10.1039/c9ra10434h)
Supplement: RA-010-C9RA10434H-s001 [file RA-010-C9RA10434H-s001.pdf]

Electronic Supplementary Material (ESI) for RSC Advances.
This journal is © The Royal Society of Chemistry 2019

Supporting Information

Diagnostically Analyzing ^{1}H NMR Spectra of Sub-types in Chaetoglobosins for Dereplication

Chen Lin,[be] Jian-chun Qin,[ce] Yong-gang Zhang,*[d] and Gang Ding*[a]

Chaetoglobsin B (**2**) = (G-type + e-type)

The Fig. **S1** is the full ^{1}H NMR spectrum of analog **2** in $CDCl_3$. The proton signals in the down-fielded zone are nearly same as that of chaetoglobosin A, which implied that this compound also contain the e-type in macrocyclic ring (Fig. **S1**). In the high-field, there are two singlet methyl groups (chemical shift values over 1.5 ppm implying their connection with double bond) suggesting the presence of a double bond C-5/C-6. In addition, a proton signal with the chemical shift value at 3.94 ppm (doublet, J = 9.5 Hz, H-7) is observed, which reveals that C-7 should possess a free hydroxyl group. This demonstrated that G-type of perhydro-isoindolone moiety must be present in this chaetoglobosin (Fig. **S2**). Thus this compound belongs to the combination of G-type and e-type, which meets the structural features of chaetoglobosin B.

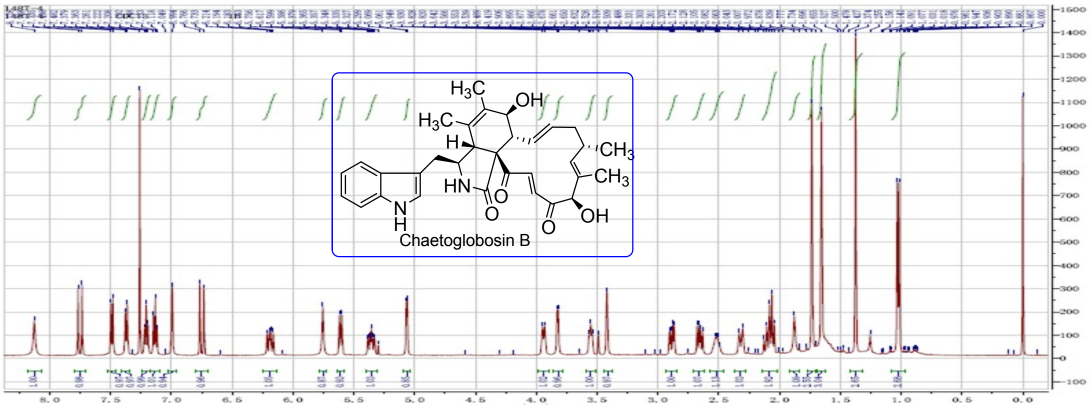

Figure S1. The full spectrum of chaetoglobosin B (**2**) in $CDCl_3$

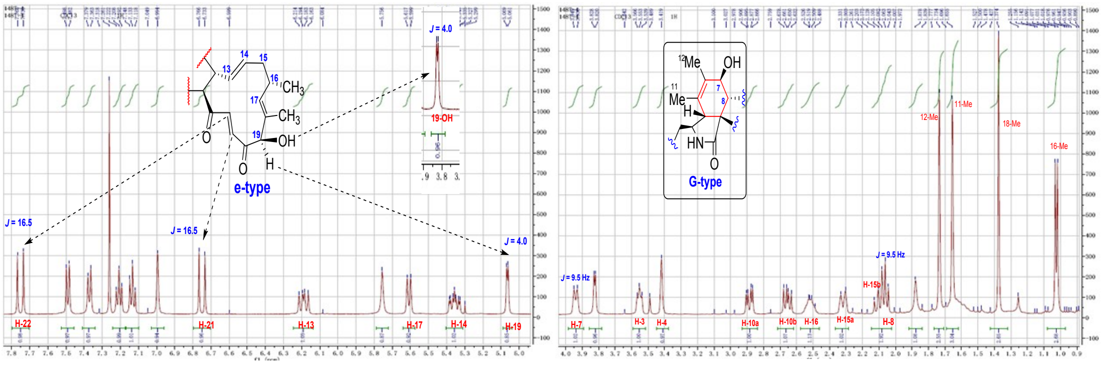

Figure S2. Expanded ^{1}H NMR spectrum of chaetoglobosin B (**2**) in $CDCl_3$

Chaetoglobosin V (**3**) = (G-type + j-type)

The ^{1}H NMR spectrum of analog **3** is done in DMSO-d_6 (Fig. **S3**). In the down-fielded zone, there are three active protons, in which the unique proton signal at 9.00 ppm (19-OH) implied the existence of j-type, and this is further supported by the disappearance of olefincia proton H-17 signal and the chemical shift value of 18-Me down-fielded to 1.90 ppm (Fig. **S4**). The H-7 as doublet peak at 4.67 (J = 8.0 Hz) together with 11-Me and 12-Me as two singlet establishes the G-type of the perhydro-isoindolone moiety. Though several proton signals are overlapped significantly in the high-fielded zone, considering the interals and chemical shift values of these protons, it meet the combination of G and j sub-types. Thus this analog was determined to be chaetoglobosin V.

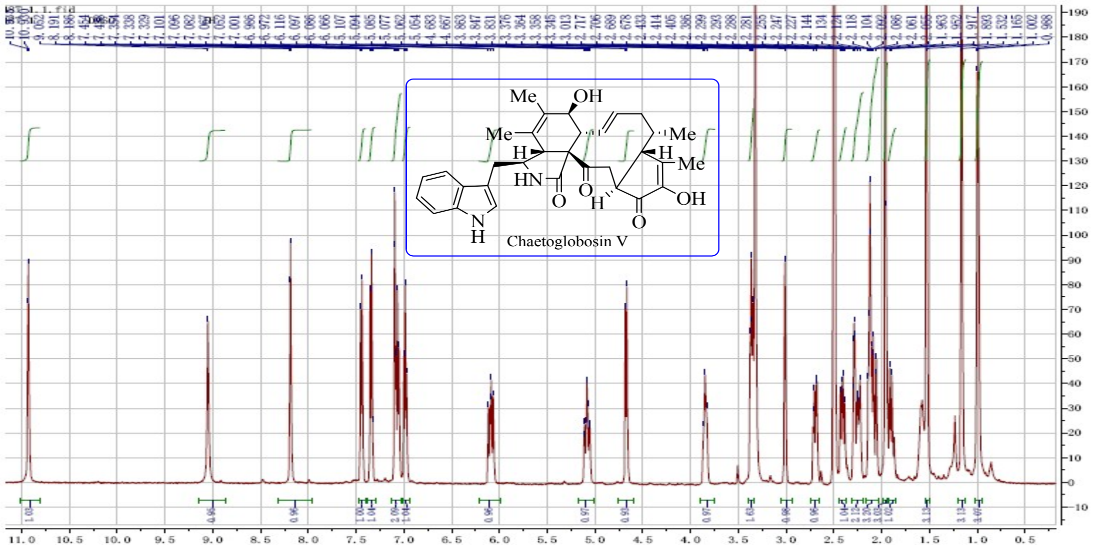

Figure S3. The full spectrum of chaetoglobosin V (**3**) in DMSO-d_6

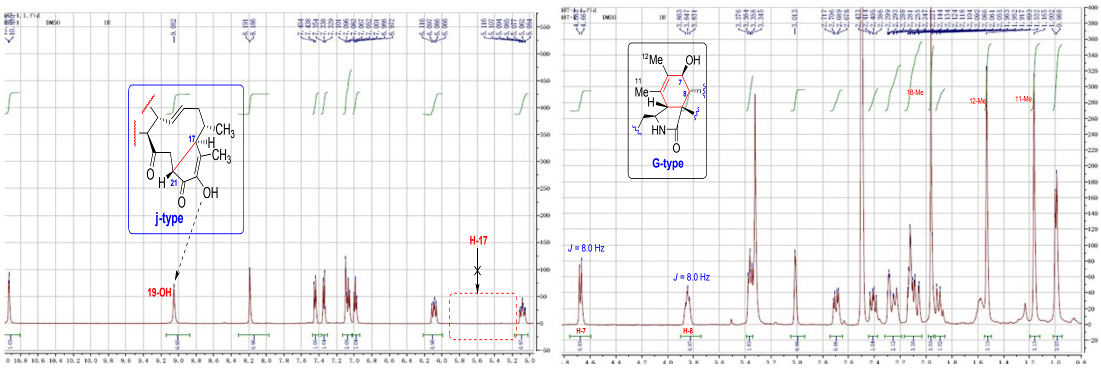

Figure S4. Expanded ^{1}H NMR spectrum of of chaetoglobosin V (**3**) in DMSO-d_6

Isochaetoglobosin D (**4**) = (E-type + i-type) (Fig. **S5**)

The ^{1}H NMR spectrum of compound **4** displayed only three methyl groups in the high field and one pair of terminal olefinic protons at 4.96 and 5.18 ppm, which implied that C-6 and C-12 must shape the corresponding double bond. The proton signal at 3.69 ppm (dd, J = 5.5, 4.5 Hz, H-7) coupled with the signal at 4.70 ppm (d, J = 5.5 Hz, 7-OH) together with the 11-Me as a doublet peak confirms that this compound possesses E-type in perhydro-isoindolone ring (Fig. **S6**). The *trans*-olefinic protons of H-21 and H-22 are not observed in down-fielded zone indicating these two carbons C-21/22 as methylene units. Considering the chemical shift value of H-17 being down-fielded to 5.86 ppm [-19C=O-18C(CH_3)=17CH] and interals in the high-field zones together with no additional oxymethine observed, this demonstrated that both C-19 and C-20 must be two keto-groups, which matches the i-type in the marocylic ring system (Fig. **S6**). Thus this compound is determined to be isochaetoglobosin D (E-type + i-type).

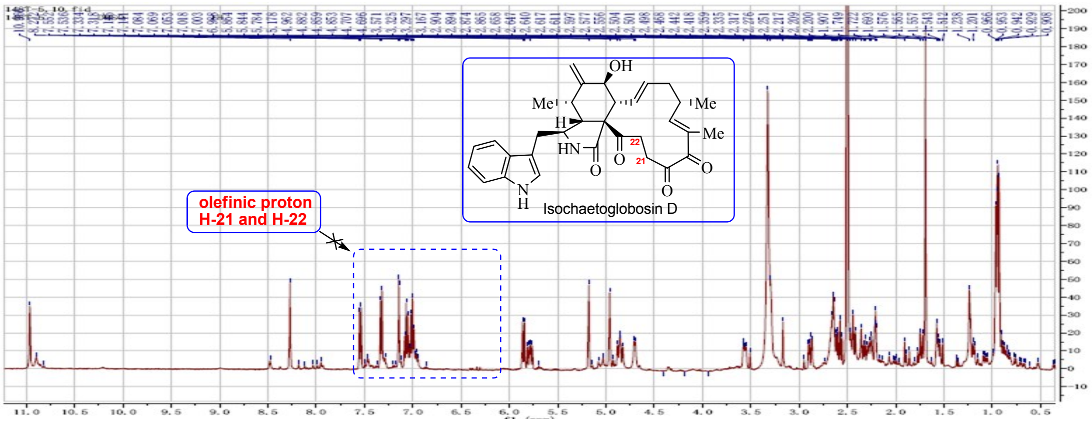

Figure S5. The full spectrum of isochaetoglobosin D in DMSO-d_6

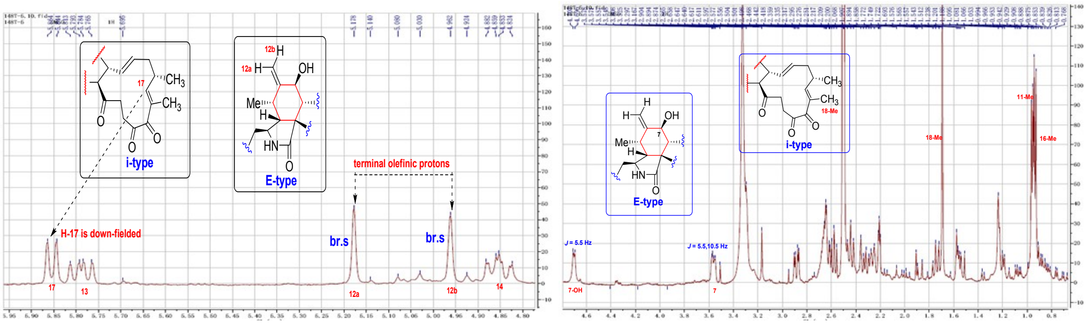

Figure S6. Expanded ^{1}H NMR spectrum of isochaetoglobosin D in DMSO-d_6

Chaetoglobosin E (**5**) = (G-type + g-type)

The ^{1}H NMR spectrum of compound **5** (in DMSO-d_6) reveals that this analog does not possess the double bond H-21/H-22 in macrocyclic ring (Fig. **S7**). The low chemical shift value of H-17 at 6.28 ppm indicates that C-19 possesses the keto-group

[-19C=O-18C(CH_3)=17CH]. The doublet doublet peak at 4.70 ppm (J = 6.5, 12.5 Hz, H-20) coupled with the doublet signal at 4.78 ppm (J = 6.5 Hz, 20-OH) suggests that C-20 contains a free hydroxyl group. This implies that g-type is found in this compound (Fig. **S8**). The big coupling constant between 4.49 ppm (doublet, H-7) and 3.62 ppm (triplet, H-8) along with the two methyls as singlet peaks (δ_H = 1.52 and 1.11 ppm) confirm the existence of G-type (Fig. **S8**). This result revealed that this analog possesses the combination of G and g types, which establish its structure as chaetoglobosin E.

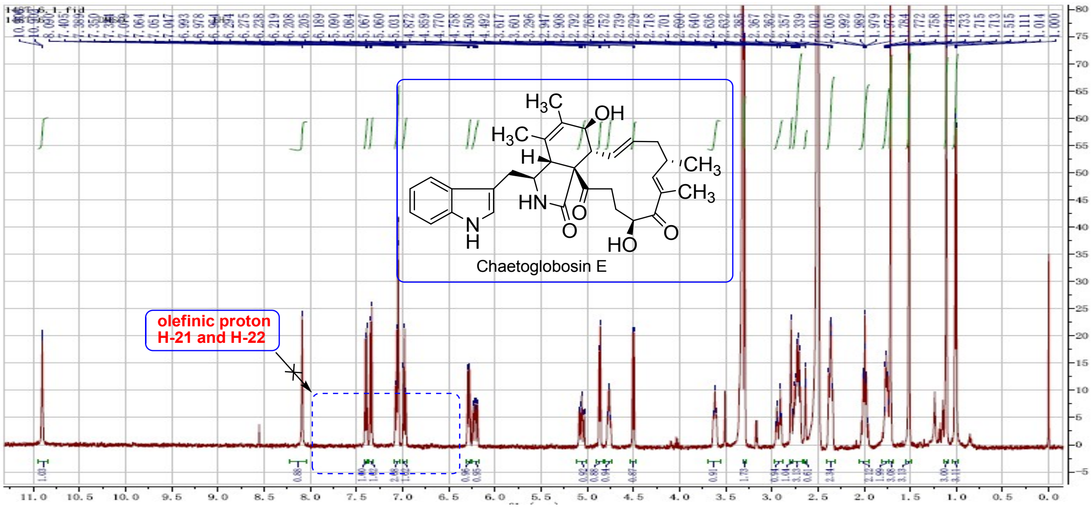

Figure S7. The full spectrum of chaetoglobosin E in DMSO-d_6

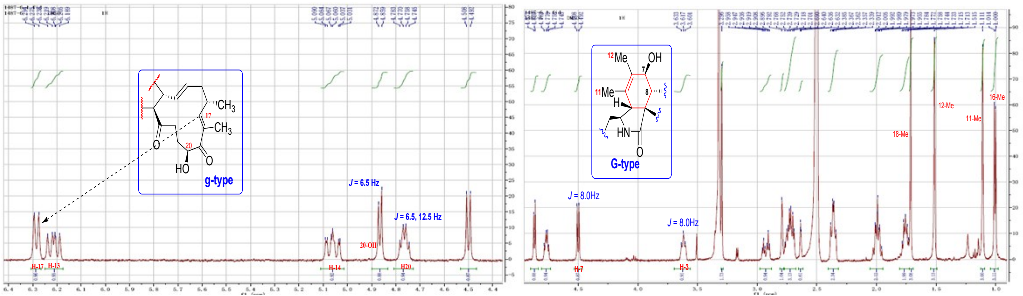

Figure S8. Expanded ^{1}H NMR spectrum of chaetoglobosin E in DMSO-d_6

Chaetoglobosin G (**6**) = (G-type + i-type)

The analog **6** do not possess the double bond C-21 and C-22 from the analysis of the down-fielded zone in the ^{1}H NMR spectrum in $CDCl_3$ (Fig. **S9**). The down-fielded chemical shift value of H-17 (6.08 ppm) and absence of 20-oxymethine proton signal in the spectrum implied i-type of the macrocyclic ring. The high-fielded zone of analog **6** implied the existence of G-type with a double bond at C-5 and C-6, and a free hydroxyl group at C-7 same as that of chaetoglobosin E (**5**) (Fig. **S10**). Thus analog **6** is determined to chaetoglobosin G on the basis of analyzing the ^{1}H NMR

spectrum.

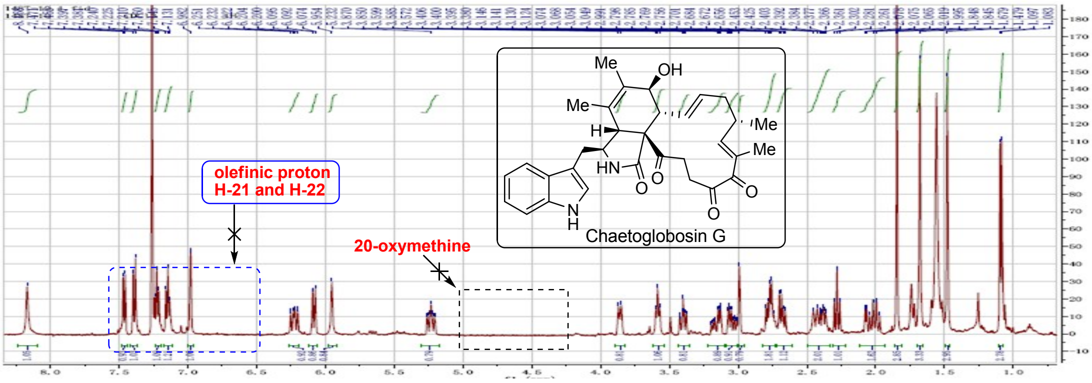

Figure S9. The full spectrum of chaetoglobosin G in $CDCl_3$

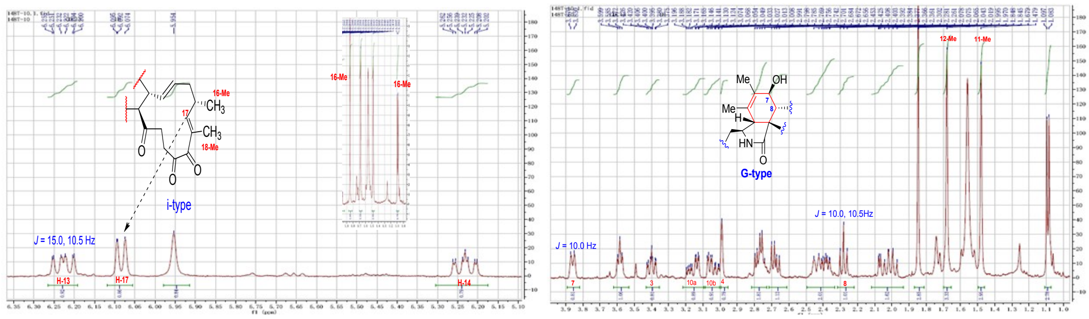

Figure S10. Expanded ^{1}H NMR spectrum of chaetoglobosin G in $CDCl_3$

Chaetoglobosin R (**7**) = (D-type + e-type)

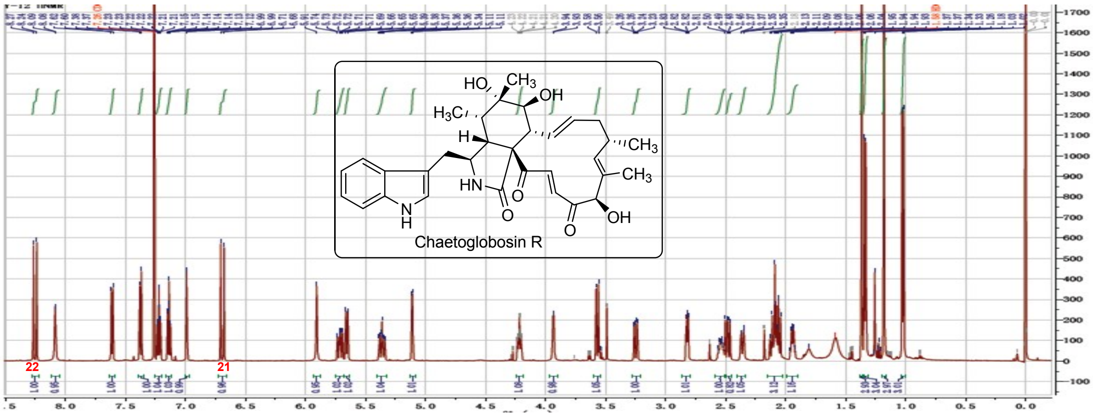

Figure S11. The full spectrum of chaetoglobosin R in $CDCl_3$

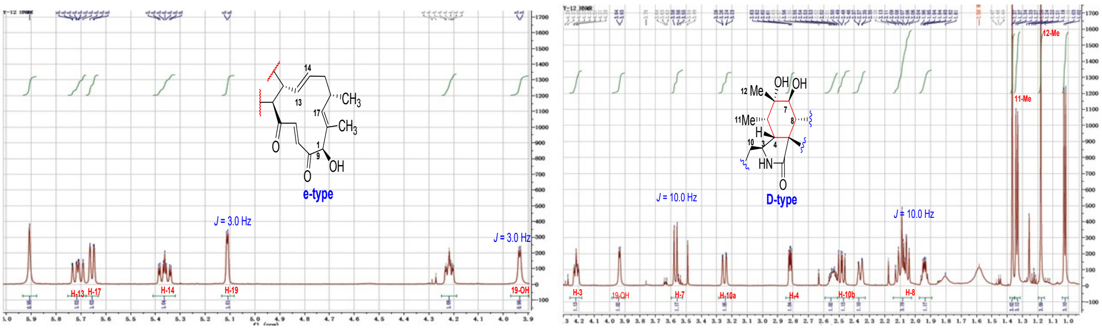

Figure S12. Expanded ^{1}H NMR spectrum of chaetoglobosin R in $CDCl_3$

The down-fielded proton signals in the analog **7** implied that this compound possesses the e-type in the macrocyclic ring system same as those of chaetoglobosins A and B (Fig. **S11**). In the high-fielded zone, there exists a singlet (12-Me) and doublet methyl (11-Me) except for the 16-Me and 18-Me, which implies that compound **7** might contain C or D-types in the perhydro-isoindolone moiety. Yet the proton signal at 3.57 ppm (doublet, $J_{7,8}$ = 10.0 Hz, H-7) coupled with the peak at 2.06 ppm (H-8) denotes that C-7 must possess a free hydroxyl group not the expoxide group at C-6 and C-7. Thus it reveals the existence of 6, 7-diol group in compound **7**, which confirms the D-type contained in this compound (Fig. **S12**). Literature searching reveals that both chaetoglobosins Q and R possess the D-type plus e-type with different stereochemistry at C-12. Comparison of the ^{1}H NMR data established the analog **7** to be chaetoglobosin R.

Prochaetoglobosin IIIed (**8**) = (G-type + d-type)

The high-fielded zone of analog **8** displays the diagnostic signals of G-type as those of chaetoglobosins B, E and G (Fig. **S13/14**). The olefinic protons of H-21 and H-22 with chemical values at 6.72 and 7.62 ppm, respectively, implied that the C-20 possesses a keto-group. The chemical shift value of H-17 is not down-fielded compared chaetoglobosins D, E and G, which demonstrates that C-19 does not contain the keto-group. In addition, two proton signals as doublets at 3.57 (H-19a) and 3.02 ppm (H-19b) with big coupling constants (J = 11.0 Hz) are observed. This speculation reveals that C-19 must be a methylene unit, which confirms that analog **8** possess d-type in the macrocylic ring system (Fig. **S13/14**). Finally, this compound (**8**) is elucidated to be prochaetoglobosin IIIed compared with literatures.

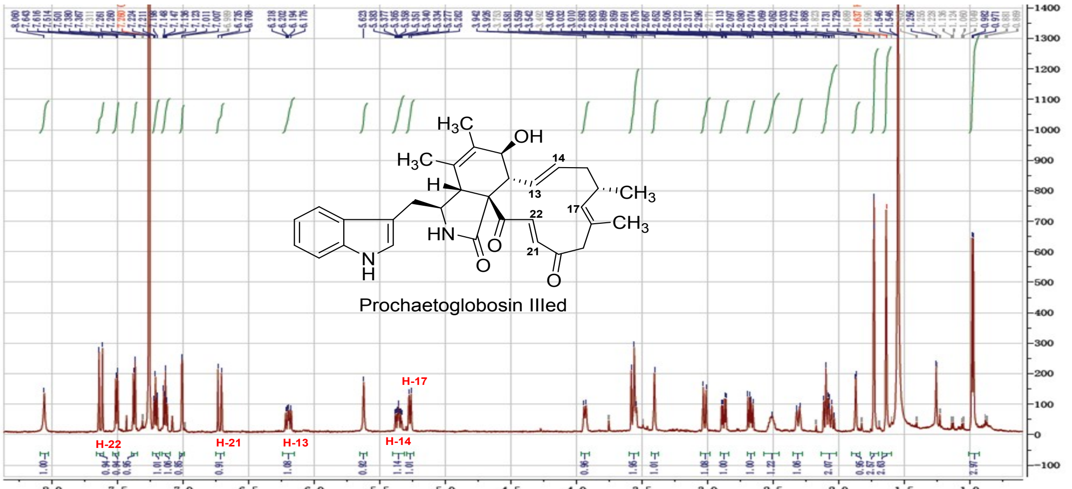

Figure S13. The analysis of ^{1}H NMR spectrum of prochaetoglobosin IIIed (**8**) in $CDCl_3$

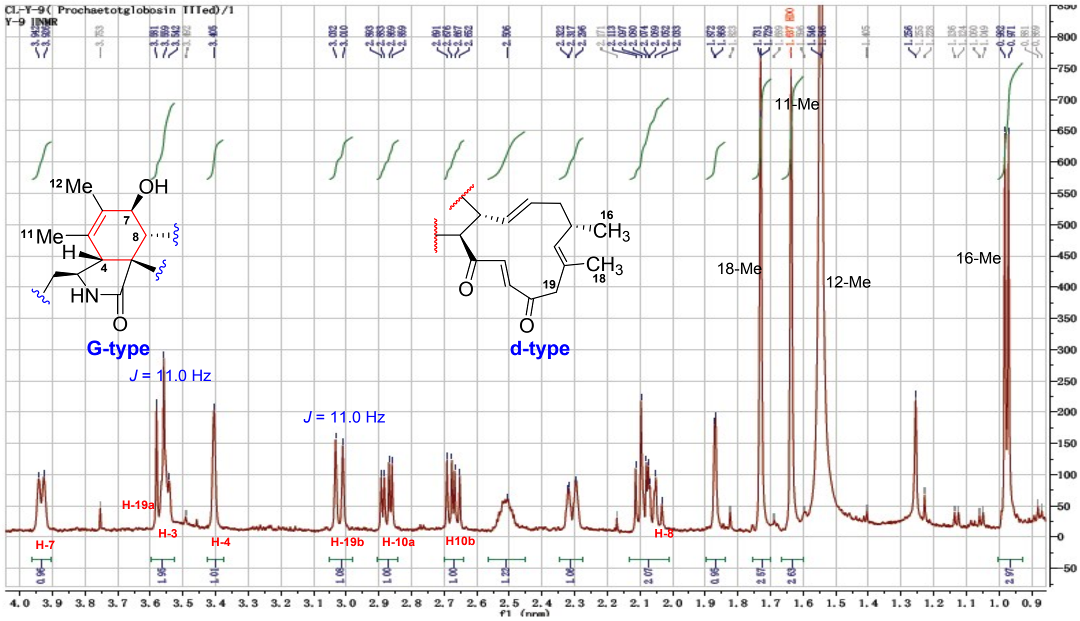

Figure S14. The analysis of ^{1}H NMR spectrum of prochaetoglobosin IIIed (**8**) in $CDCl_3$

Chaetoglobosin D (**9**) = (E-type + e-type)

The ^{1}H NMR spectrum of **9** displayed the e-type in the macro-ring system including the diagnostic signals: the α, β-unsaturated keto group (C-20-C21-C22-C23), 19-CH, 19-OH, olefinic protons H-13, H-14 and H-17 together with 16-Me and 18-Me (Fig. **S15**). The typically terminal protons (C=CH_2 at 5.00 ppm), 7-CH (3.68, dd, J = 9.6, 6.6 Hz), 7-OH (4.76, d, J = 6.6 Hz), and 8-H (2.38, t, J = 9.6 Hz) and the doublet signal of 11-Me implied that compound **9** possessed the E-type of perhydro-isoindolone moiety (Fig. **S15**). Thus this compound was determined to be the combination of E-type and e-type, which matches the structural features of chaetoglobosin D.

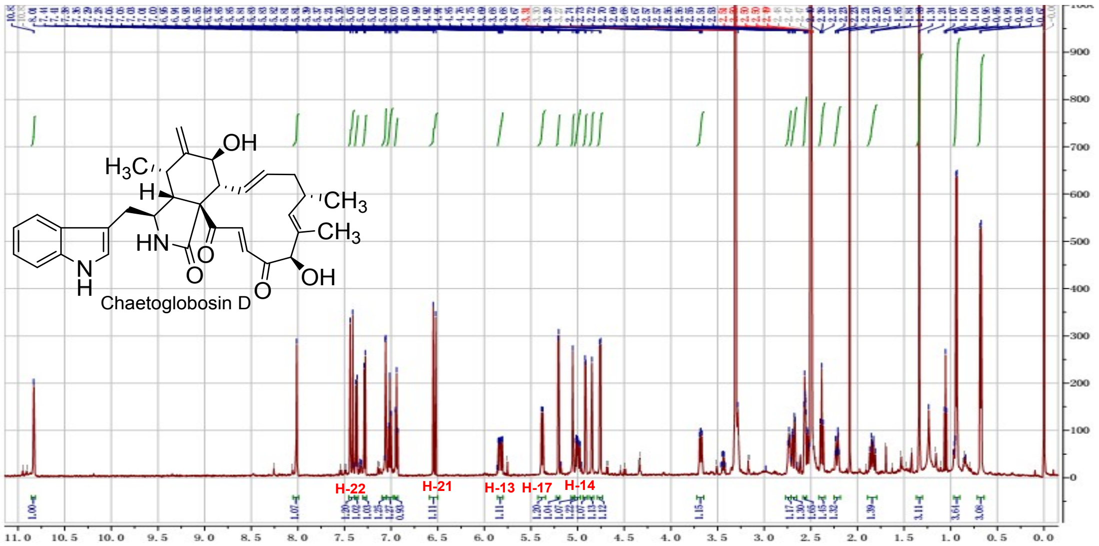

Figure S15. The full spectrum of chaetoglobosin D (**9**) in DMSO-d_6

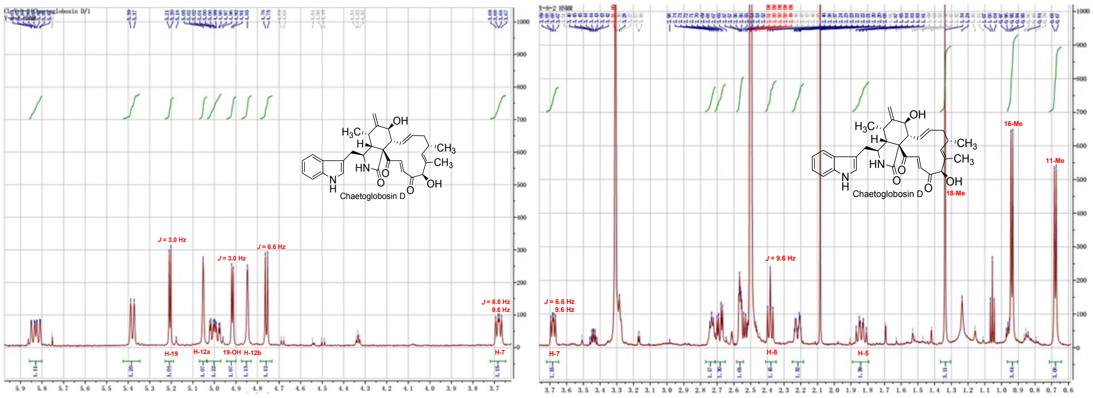

Figure S16. Expanded ^{1}H NMR spectrum of chaetoglobosin D (**9**) in DMSO-d_6

1. Structures of A–I sub-types of perhydro-isoindolone moiety (56 analogs)

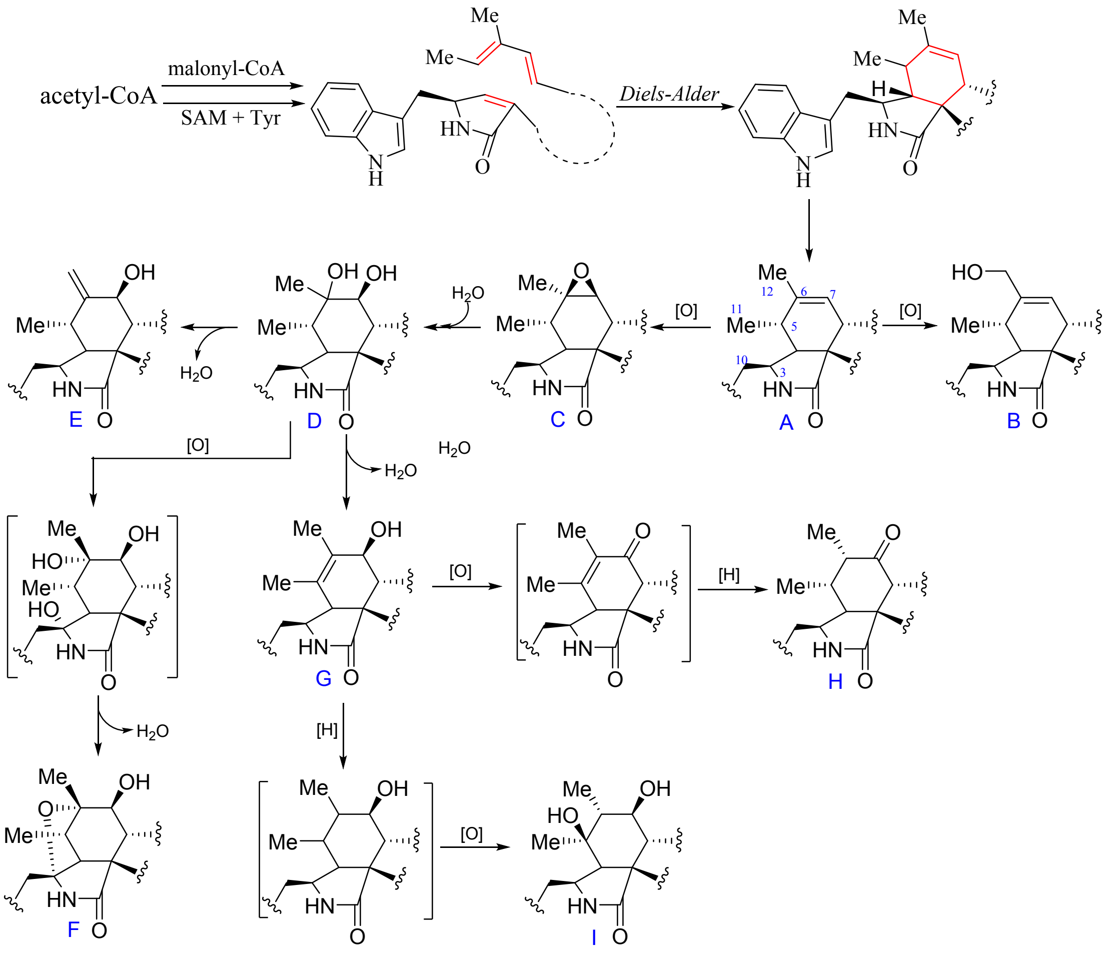

A-Type:

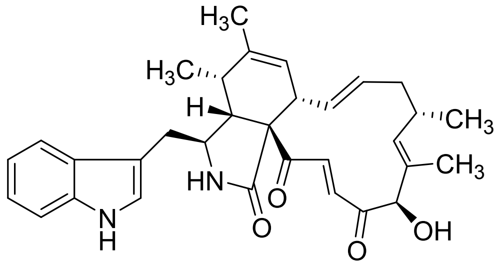

Chaetoglobosin J

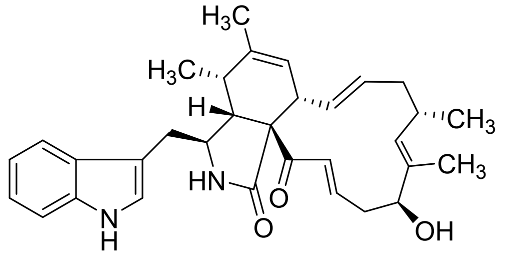

Chaetoglobosin T

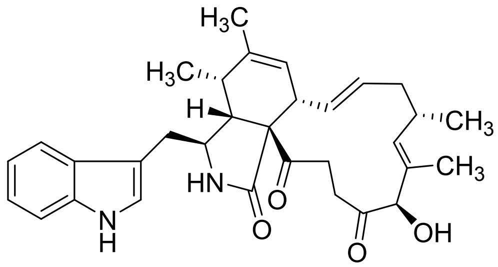

Penochalasin G

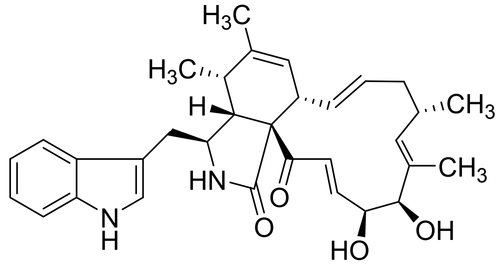

Cytoglobosin D

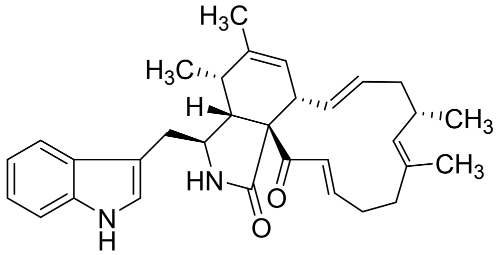

prochaetoglobosin I

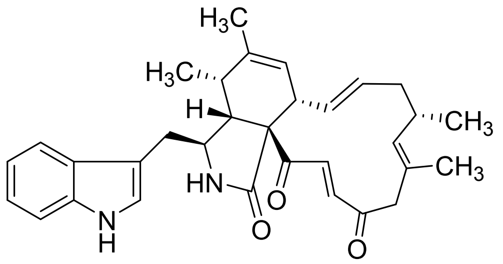

prochaetoglobosin II

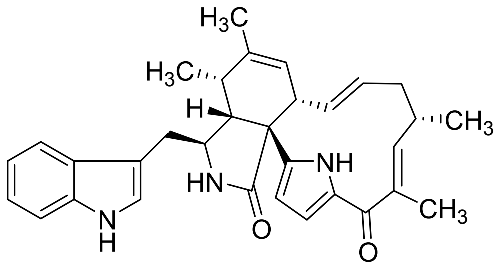

Armochaetoglobin K

B-type:

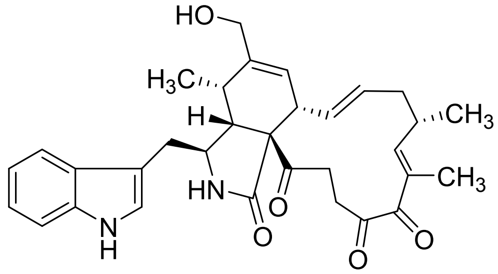

Cytoglobosin E

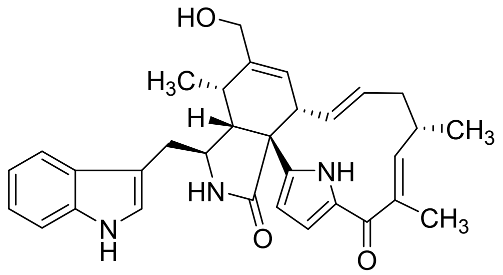

Armochaetoglobin L

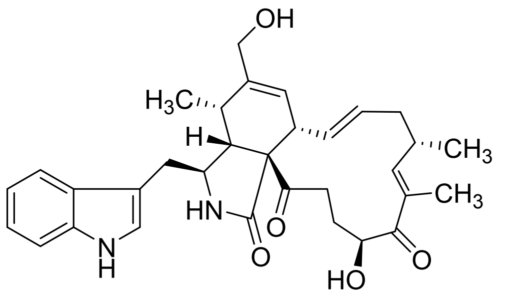

armochaetoglobin W

C-type:

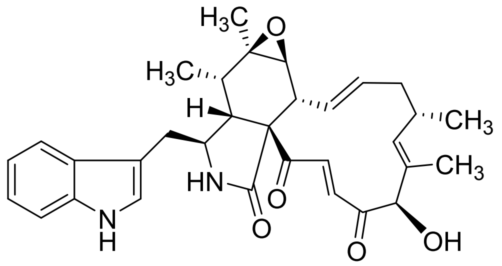

Chaetoglobosin A

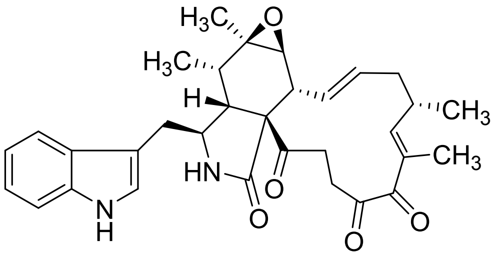

Chaetoglobosin C

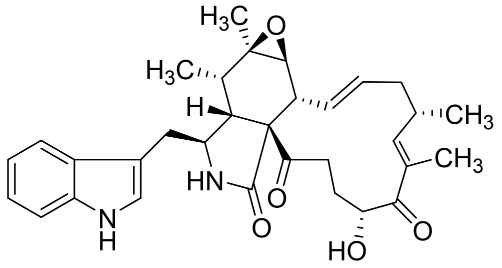

Chaetoglobosin F

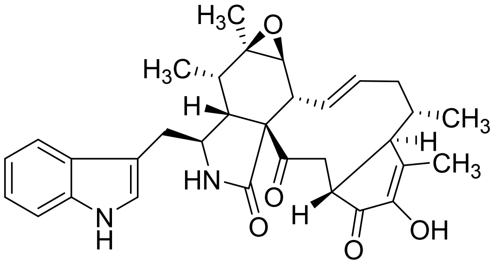

Chaetoglobosin U

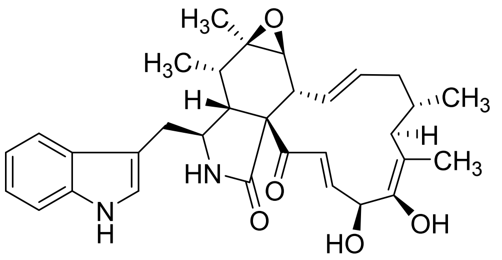

20-dihydrochaetoglobosin A

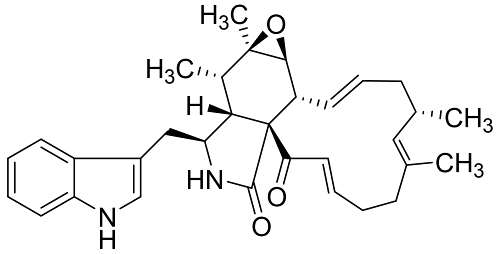

Prochaetoglobosin IV

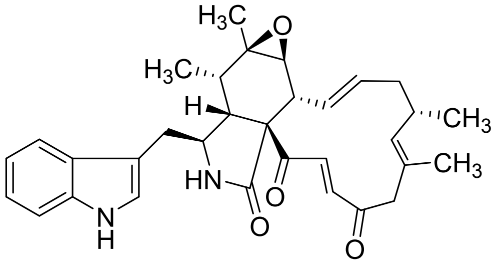

Prochaetoglobosin III

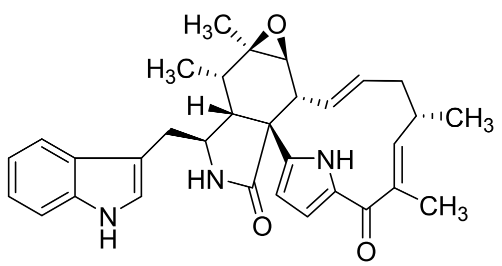

Penochalasin A

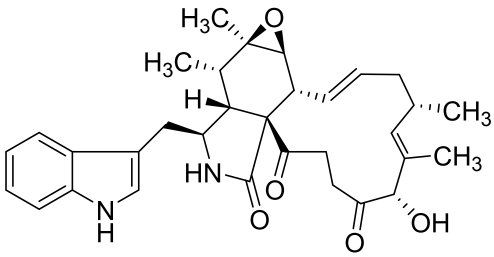

Penochalasin E

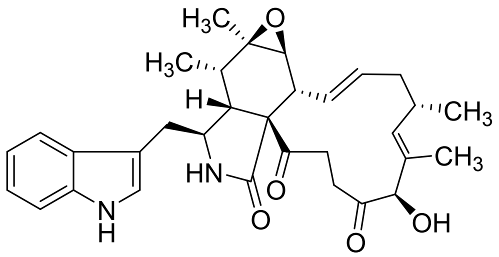

Penochalasin F

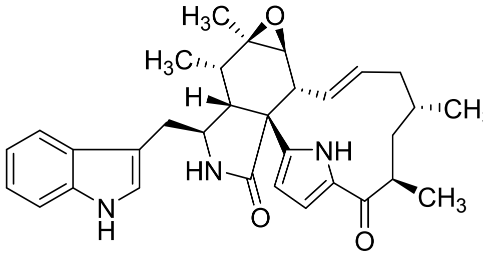

Armochaetoglobin P

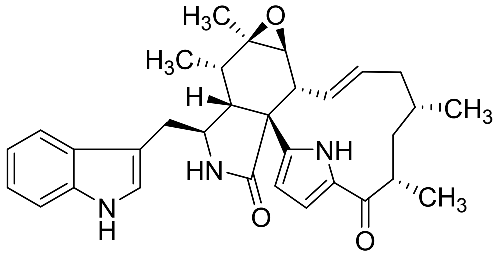

Armochaetoglobin Q

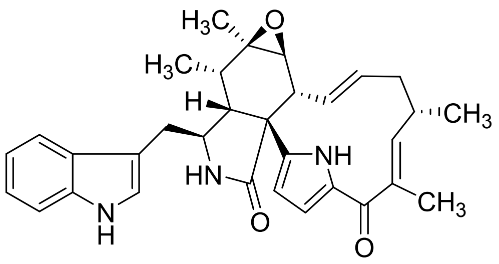

Penochalasin D

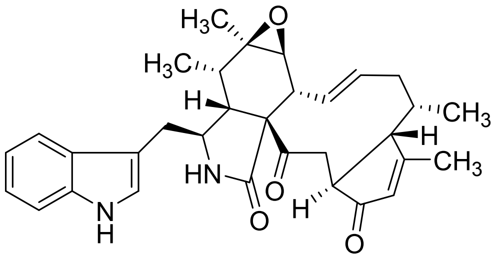

armochaetoglobin Y

D-type:

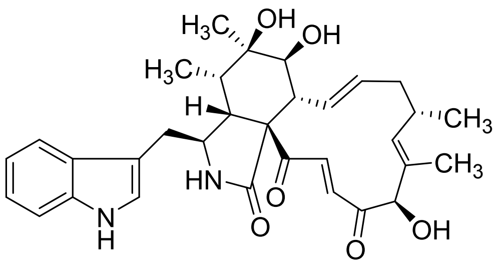

Chaetoglobosin Q

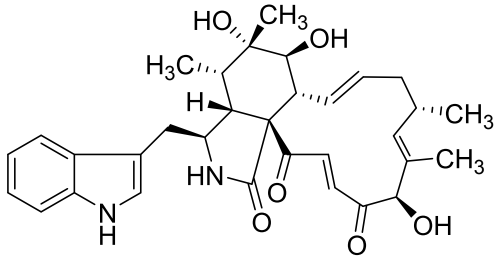

Chaetoglobosin R

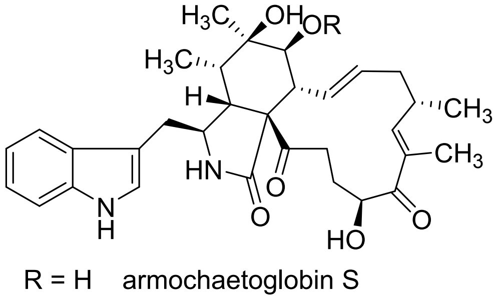

R = H armochaetoglobin S
R = Ac 7-O-acetylarmochaetoglobin S

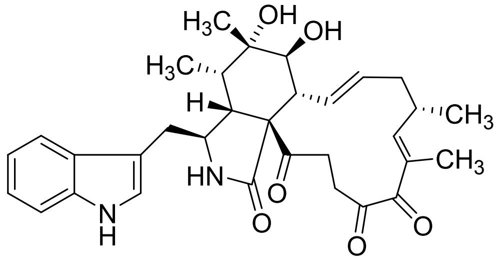

armochaetoglobin U

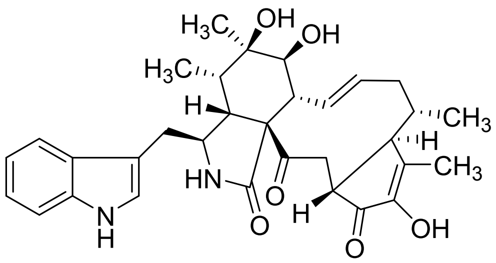

armochaetoglobin X

E-type:

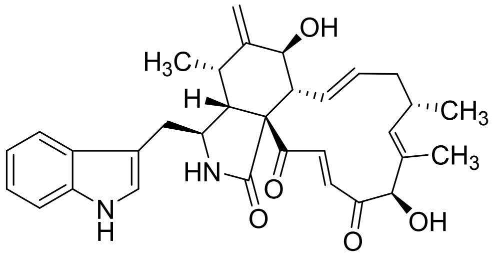

Chaetoglobosin D

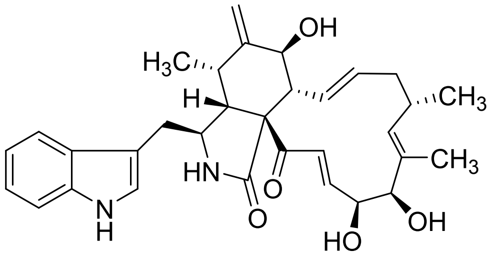

Cytoglobosin B

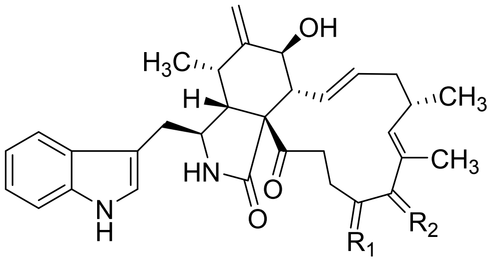

Cytoglobosin F R_1 = *O*, R_2 = H, *β*-OH
Cytoglobosin G R_1 = R_2 = H, *β*-OH
Isochaetoglogosin D R_1 = R_2 = O

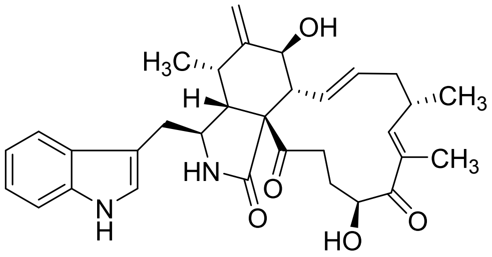

Chaetoglobosin F_{ex}

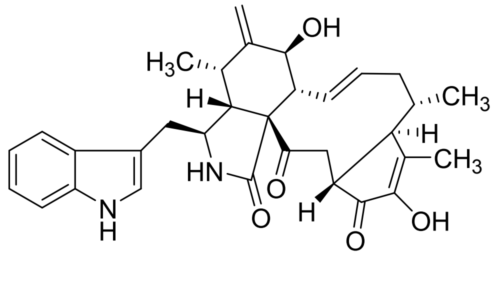

Cytoglobosin A

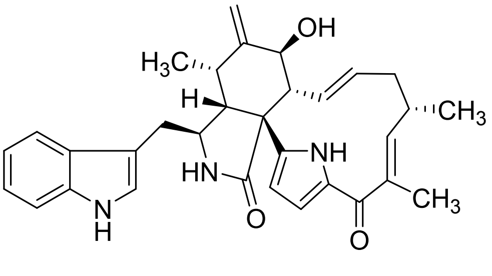

Penochalasin C

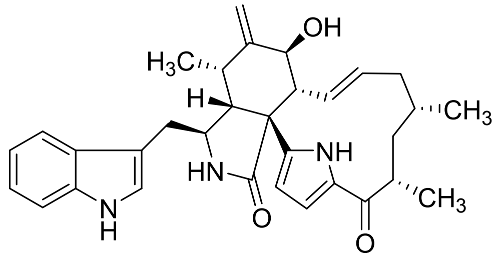

Armochaetoglobin R

F-type

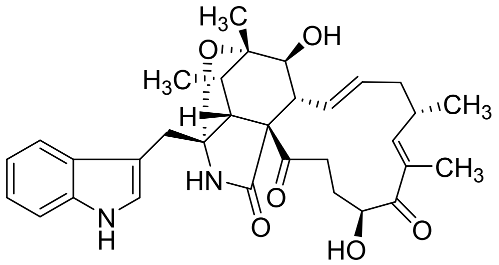

Chaetoglobosin W

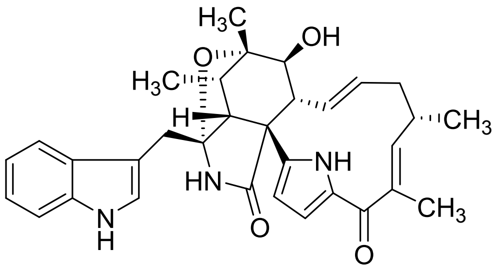

Armochaetoglobin M

G-type:

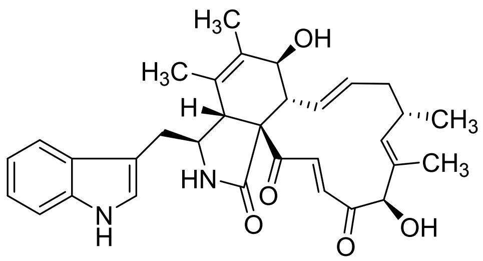

Chaetoglobosin B

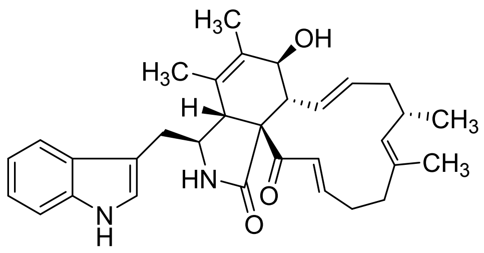

ChaetoglobosinV_2

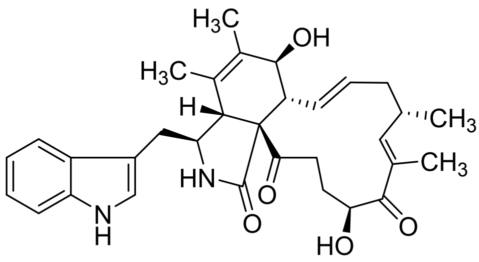

Chaetoglobosin E

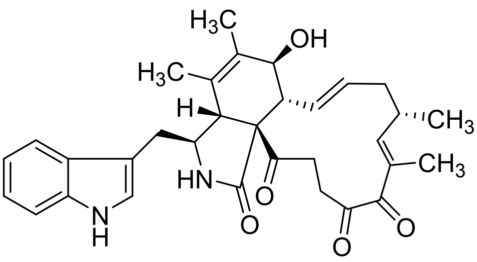

Chaetoglobosin G

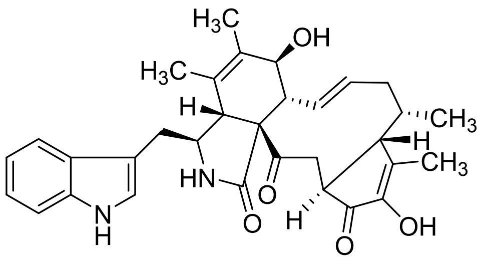

Chaetoglobosin V_1

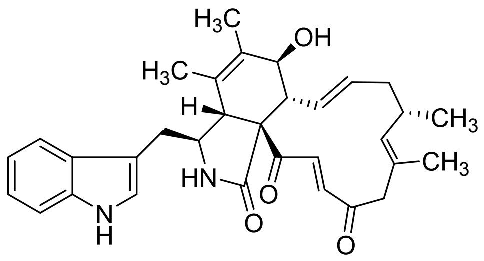

Prochaetoglobosin IIIed

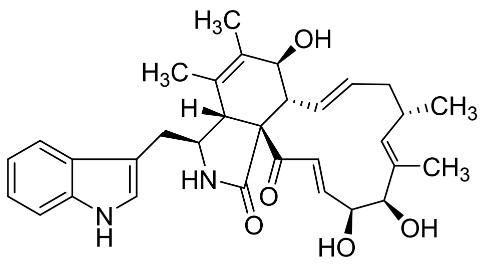

Cytoglobosin C

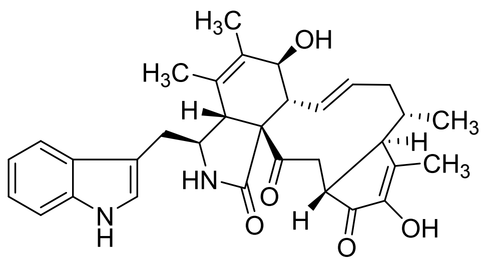

Chaetoglobosin Vb

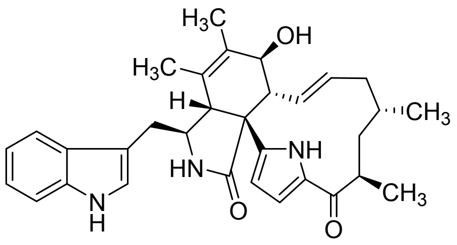

Armochaetoglobin N

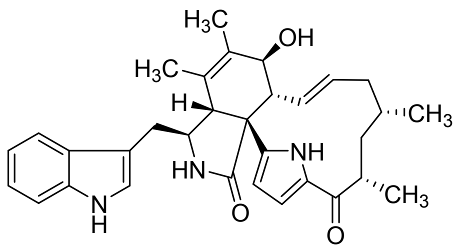

Armochaetoglobin O

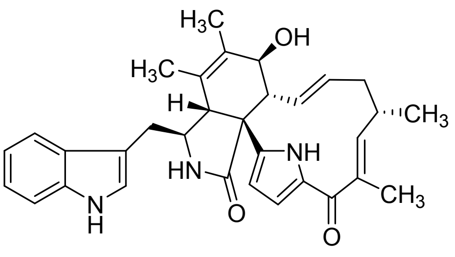

Penochalasin B

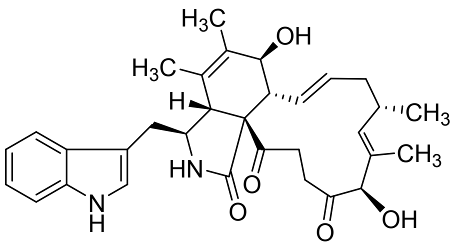

Chaetoglobosin O

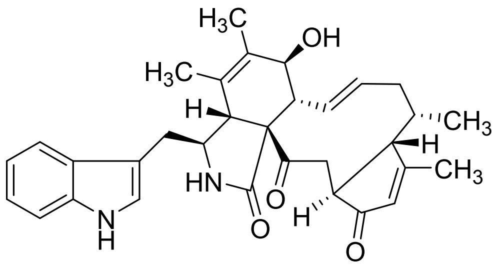

armochaetoglobin Z

H-type:

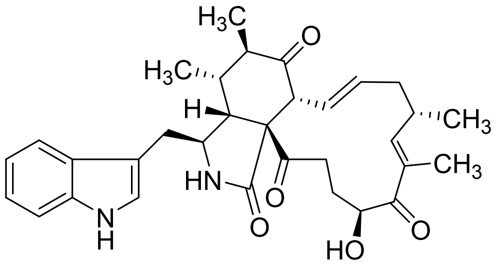

Chaetoglobosin Y

I-type:

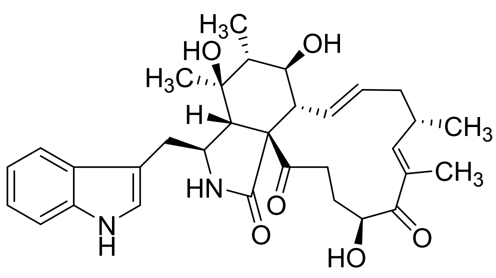

armochaetoglobin V

2. Structures of 14 sub-types (a–n types) of macrocyclic ring (56 analogs)

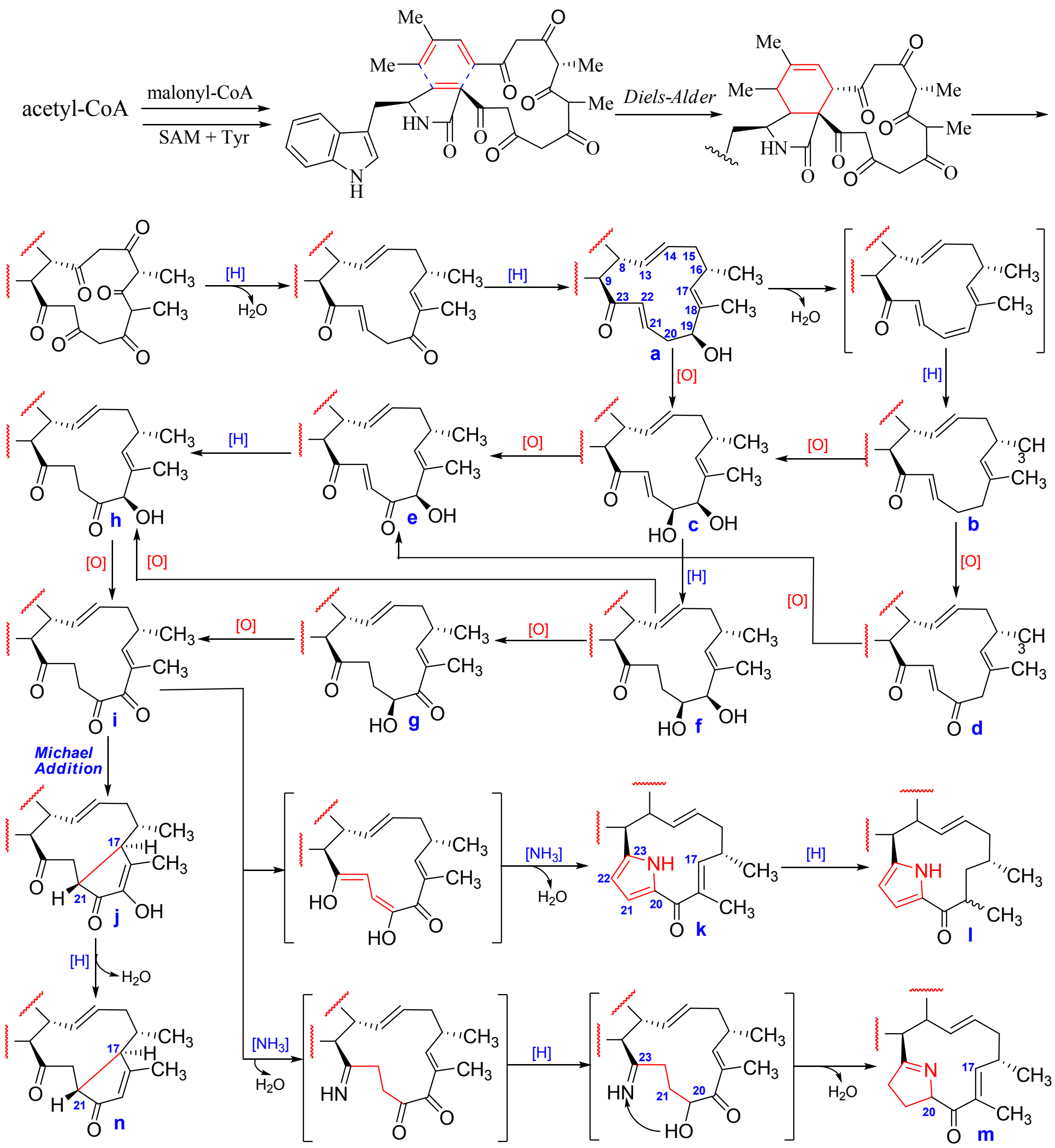

a-TYPE

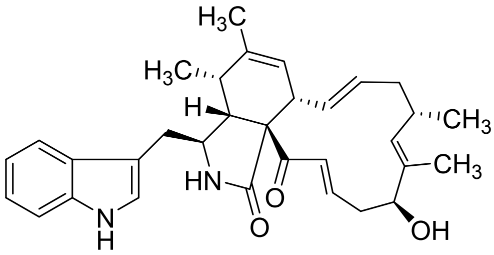

Chaetoglobosin T

b-TYPE

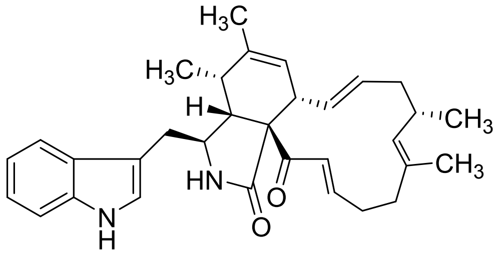

Prochaetoglobosin I

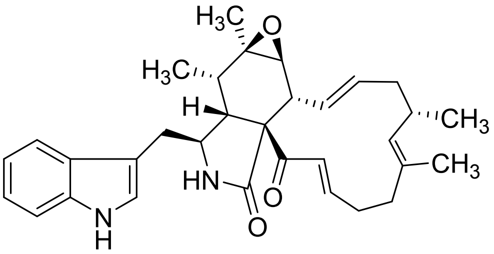

Prochaetoglobosin IV

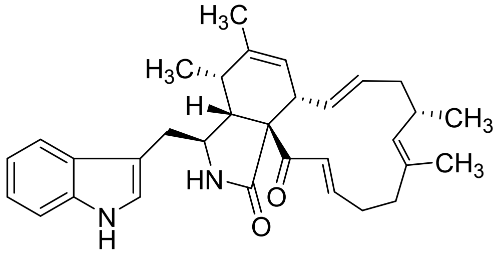

chaetoglobosin V_2

c-TYPE

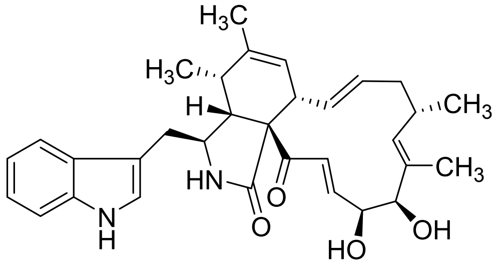

Cytoglobosin D

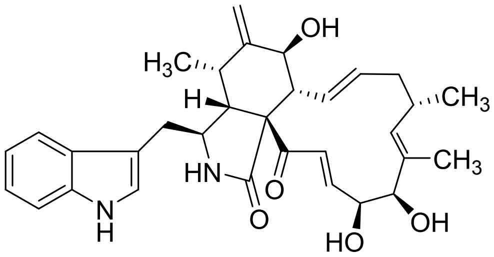

Cytoglobosin B

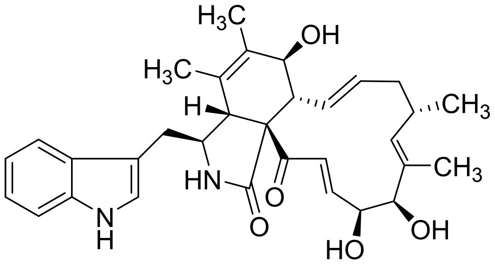

Cytoglobosin C)

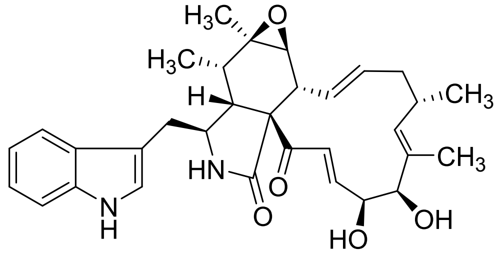

20-dihydrochaetoglobosin A

d-TYPE

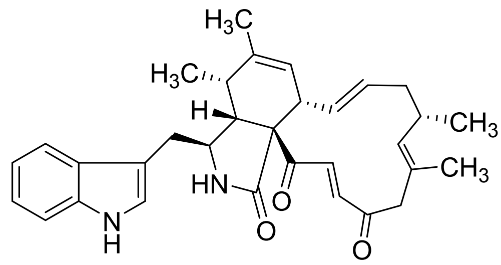

Prochaetoglobosin II

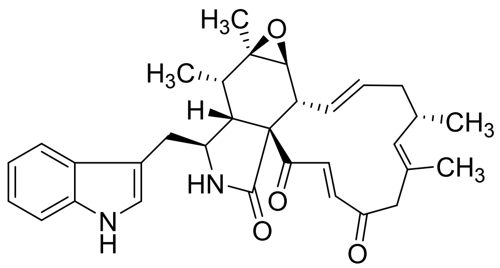

Prochaetoglobosin III

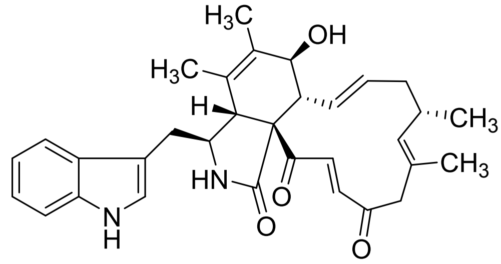

Prochaetoglobosin III_{ed}

e-TYPE

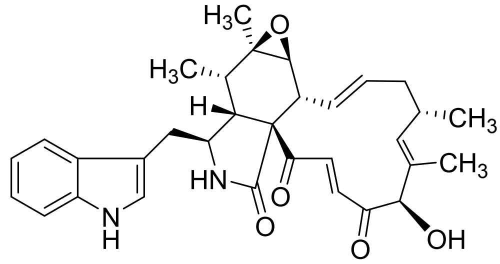

Chaetoglobosin A

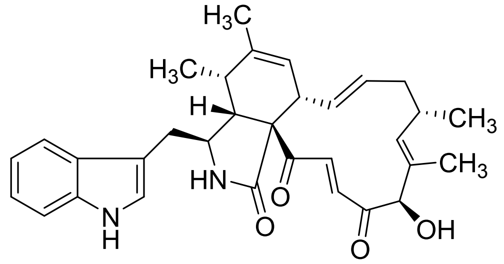

Chaetoglobosin J

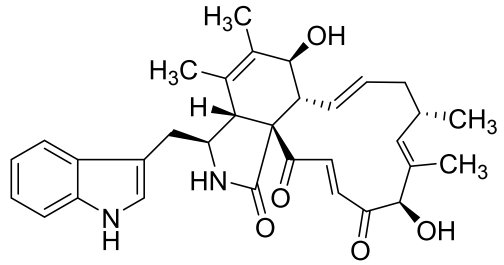

Chaetoglobosin B

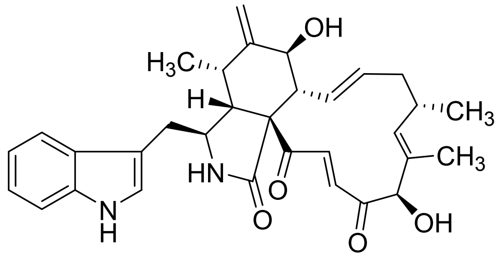

Chaetoglobosin D

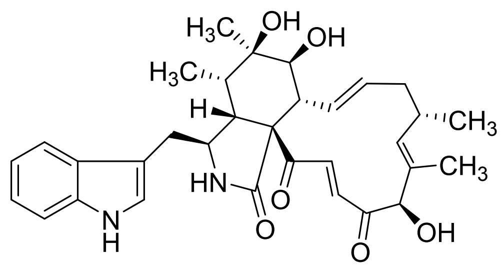

Chaetoglobosin Q

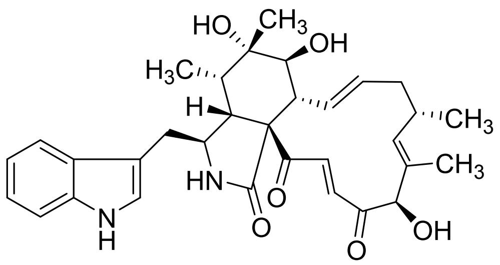

Chaetoglobosin R

f-TYPE

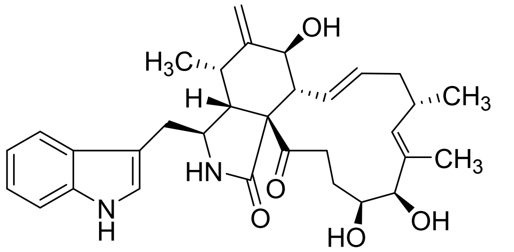

Cytoglobosin G

g-TYPE

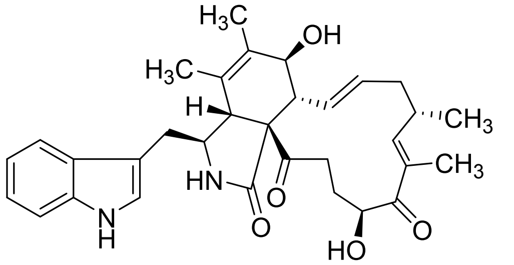

Chaetoglobosin E

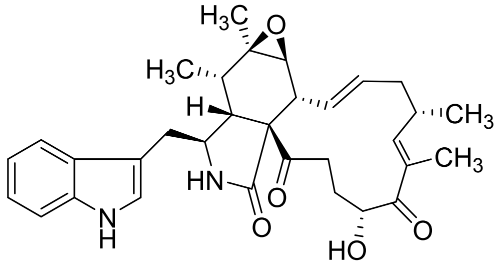

Chaetoglobosin F

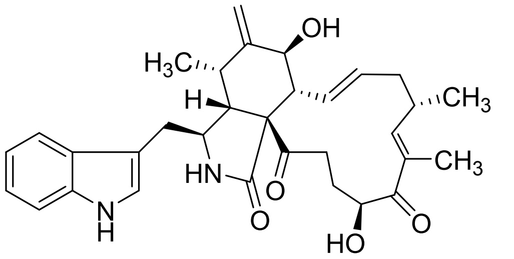

Chaetoglobosin F_{ex}

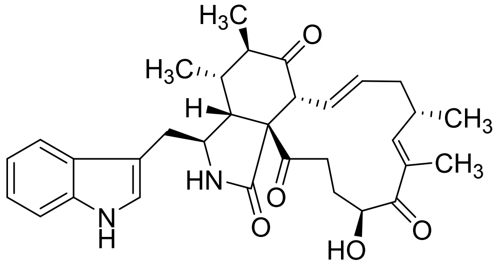

Chaetoglobosin Y

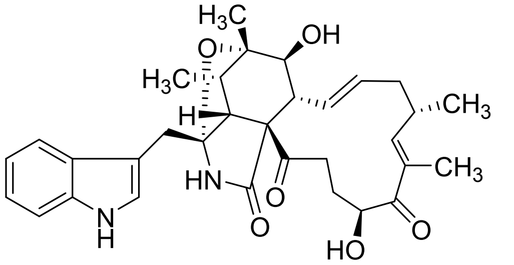

Chaetoglobosin W

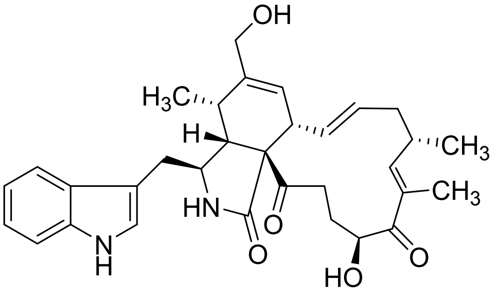

armochaetoglobin W

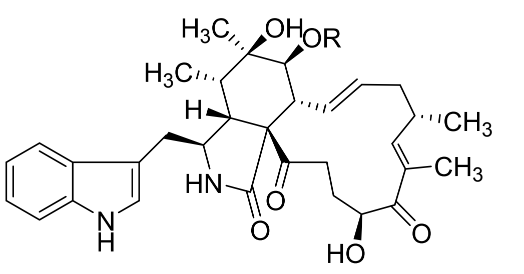

R = H armochaetoglobin S
R = Ac 7-O-acetylarmochaetoglobin S

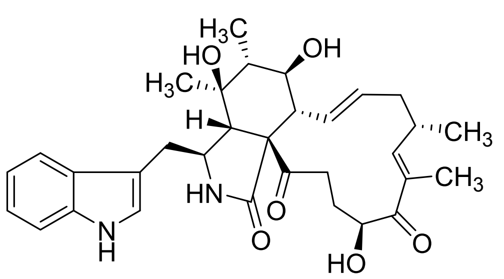

armochaetoglobin V

h-TYPE

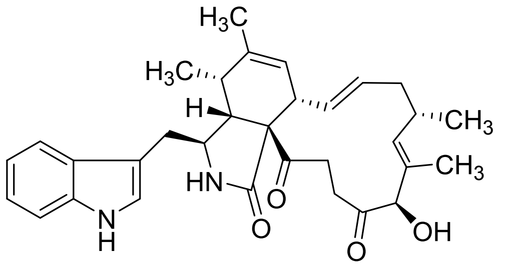

Penochalasin G

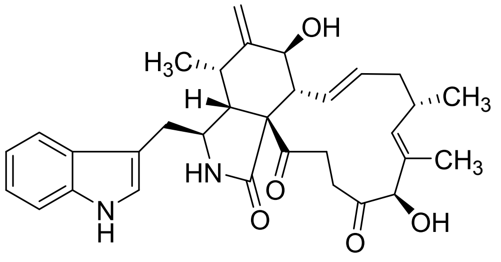

Cytoglobosin F

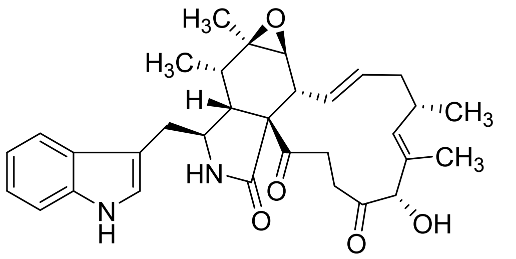

Penochalasin E

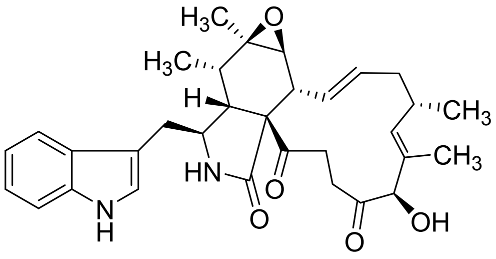

Penochalasin F

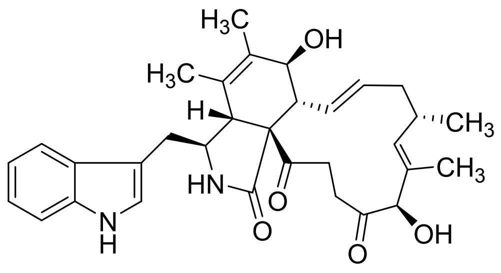

Chaetoglobosin O

i-TYPE

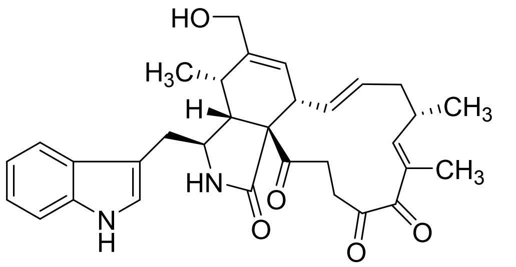

Cytoglobosin E

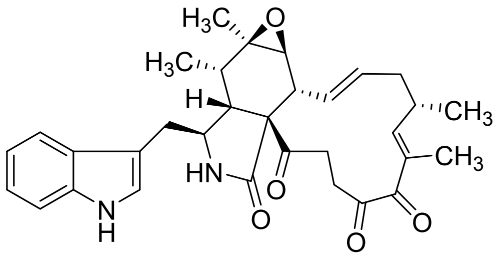

Chaetoglobosin C

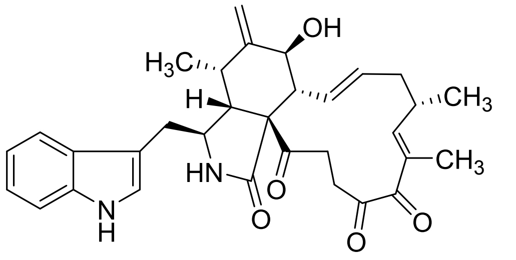

Isochaetoglogosin D

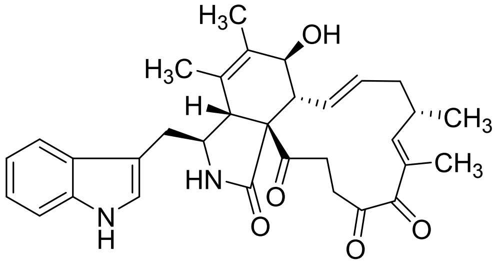

Chaetoglobosin G

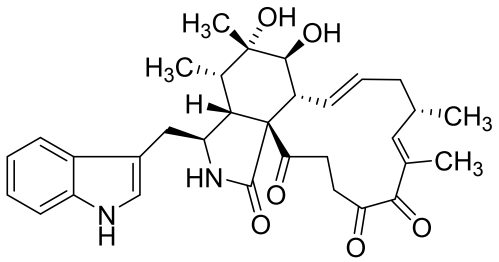

armochaetoglobin U

j-TYPE

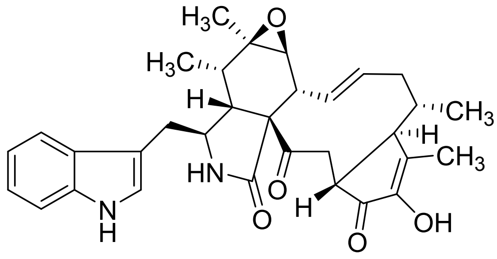

Chaetoglobosin U

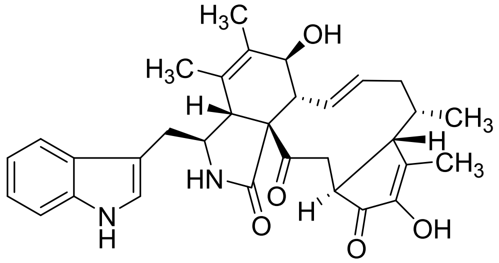

Chaetoglobosin V

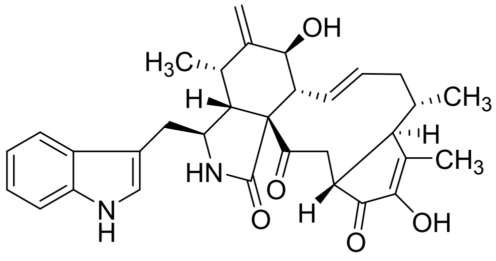

Cytoglobosin A

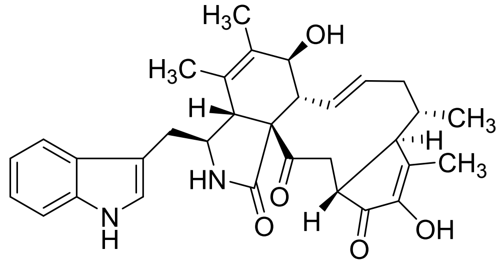

Chaetoglobosin Vb

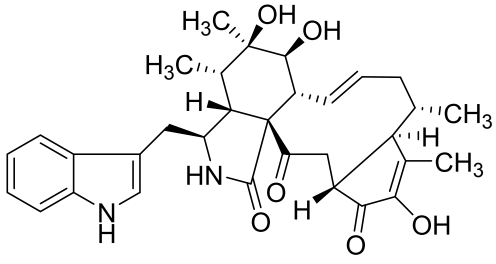

armochaetoglobin X

k-TYPE

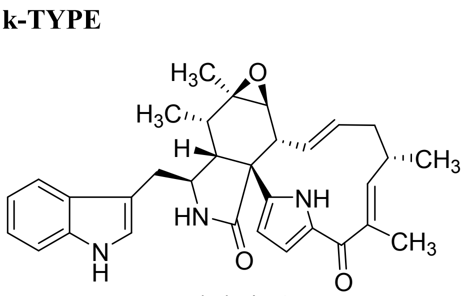

Penochalasin A

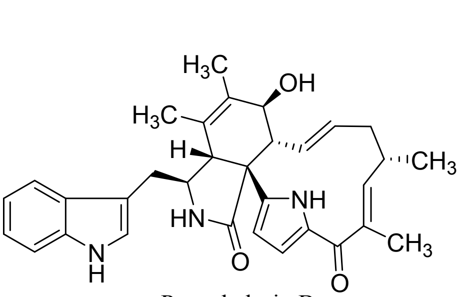

Penochalasin B

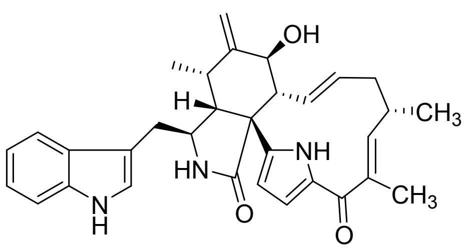

Penochalasin C

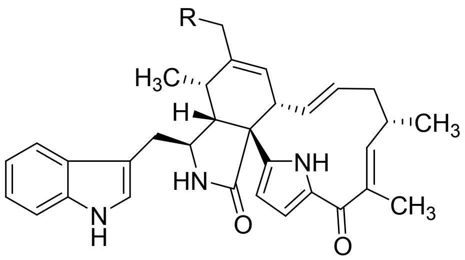

Armochaetoglobin K R = H
Armochaetoglobin L R = OH

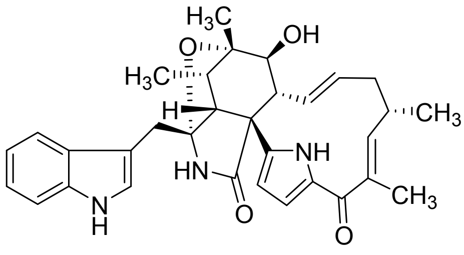

Armochaetoglobin M

l-TYPE

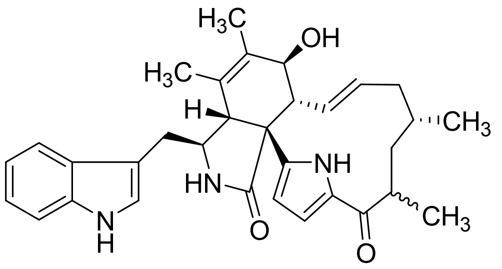

Armochaetoglobin N R = β-CH_3
Armochaetoglobin O R = α-CH_3

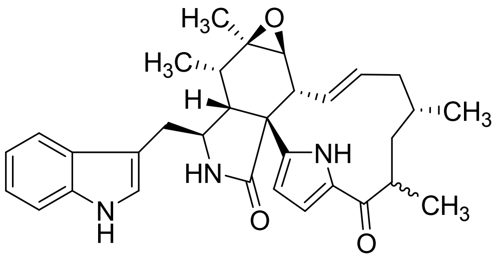

Armochaetoglobin P R = β-CH_3
Armochaetoglobin Q R = α-CH_3

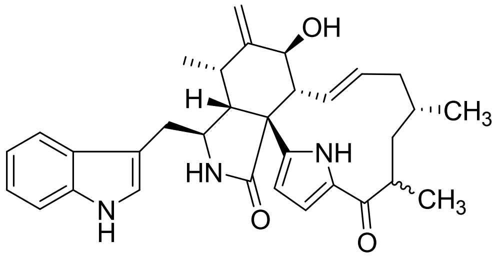

Armochaetoglobin R R = α-CH_3

m-TYPE

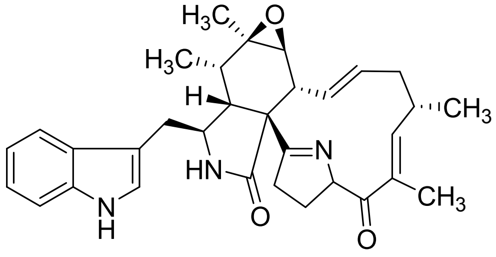

Penochalasin D

n-type:

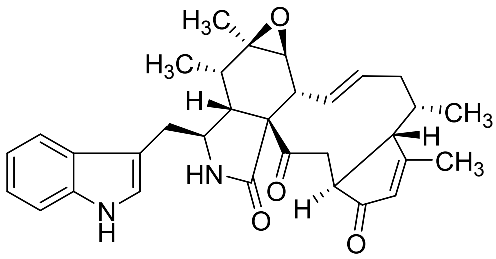

armochaetoglobin Y

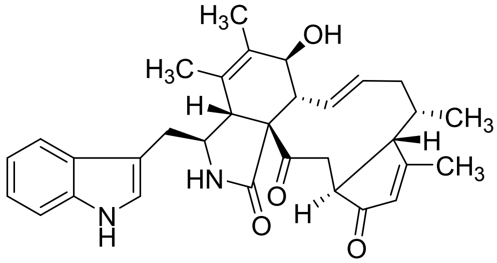

armochaetoglobin Z
